# BRCA1 Regulates Follistatin Function in Ovarian Cancer and Human Ovarian Surface Epithelial Cells

**DOI:** 10.1371/journal.pone.0037697

**Published:** 2012-06-01

**Authors:** Tejaswita M. Karve, Anju Preet, Rosie Sneed, Clara Salamanca, Xin Li, Jingwen Xu, Deepak Kumar, Eliot M. Rosen, Tapas Saha

**Affiliations:** 1 Department of Oncology, Lombardi Comprehensive Cancer Center, Georgetown University Medical Center, Washington, D.C., United States of America; 2 Department of Biochemistry and Cellular and Molecular Biology, Georgetown University School of Medicine, Washington, D.C., United States of America; 3 University of District of Columbia, Washington, D.C., United States of America; 4 Canadian Ovarian Tissue Bank, BC Cancer Research Centre, Vancouver, B.C., Canada; 5 Department of Biostatistics, Bioinformatics and Biomathematics, Georgetown University School of Medicine, Washington, D.C., United States of America; Virginia Commonwealth University, United States of America

## Abstract

Follistatin (FST), a folliculogenesis regulating protein, is found in relatively high concentrations in female ovarian tissues. FST acts as an antagonist to Activin, which is often elevated in human ovarian carcinoma, and thus may serve as a potential target for therapeutic intervention against ovarian cancer. The breast cancer susceptibility gene 1 *(BRCA1*) is a known tumor suppressor gene in human breast cancer; however its role in ovarian cancer is not well understood. We performed microarray analysis on human ovarian carcinoma cell line SKOV3 that stably overexpress wild-type BRCA1 and compared with the corresponding empty vector-transfected clones. We found that stable expression of BRCA1 not only stimulates FST secretion but also simultaneously inhibits Activin expression. To determine the physiological importance of this phenomenon, we further investigated the effect of cellular BRCA1 on the FST secretion in immortalized ovarian surface epithelial (IOSE) cells derived from either normal human ovaries or ovaries of an ovarian cancer patient carrying a mutation in *BRCA1* gene. Knock-down of *BRCA1* in normal IOSE cells demonstrates down-regulation of FST secretion along with the simultaneous up-regulation of Activin expression. Furthermore, knock-down of *FST* in IOSE cell lines as well as SKOV3 cell line showed significantly reduced cell proliferation and decreased cell migration when compared with the respective controls. Thus, these findings suggest a novel function for BRCA1 as a regulator of FST expression and function in human ovarian cells.

## Introduction

Ovarian cancer is one of the leading gynecological cancers in the United States with 22,280 estimated new cases and 15,500 deaths in 2012 (http://www.cancer.gov/cancertopics/types/ovarian; Assessed on February, 2012). High mortality rates for ovarian cancer are mainly attributed to the late stage diagnosis of disease; almost 60–65% of ovarian cancer cases are diagnosed when cancer has already metastasized beyond the confines of the ovarian tissue. Early detection of ovarian cancer is shown significantly increase patient life expectancy to as high as 85% [1]. Thus, there is a need to develop biomarkers that can be helpful in detecting ovarian cancer in early stages of the disease. Most ovarian cancers occur within the ovarian surface epithelium (OSE) and hence investigations using OSE cells derived from both normal individual and ovarian cancer patients are critical to elucidate the etiology of human ovarian cancer. Interestingly, only 5–10% of women with ovarian cancer have inherited genetic mutations in tumor suppressor genes such as *BRCA1* and *BRCA2* that predisposes them to breast and ovarian cancer [2], [3], [4]. In addition, a genetic linkage cohort study consisting of 214 breast cancer and breast-ovary cancer families combined, revealed that 90% of the patients harbored mutations in their *BRCA1* gene [5]. Moreover, *BRCA1* mutation(s) carrier females have about 15 fold greater risk for developing ovarian cancer when compared to their non-carrier female counterparts [6], [7].

Follistatin (FST), an autocrine single chain glycoprotein, is expressed in nearly all human tissues such as kidney, brain, uterus, and breast with the highest concentration found in human ovarian tissue [8]. Mature, secreted form of FST protein exists in three isoforms; full length, intermediate and shortest consisting of 315, 303 and 288 amino acids respectively [9]. FST, initially isolated from follicular fluid was found to interact with Activin, a member of the transforming growth factor-β (TGF-β) superfamily. Activin has been shown to regulate cell proliferation, differentiation, angiogenesis, as well as apoptosis, and thus may be possibly involved in regulating ovarian tumor growth [10]. Elevated levels of Activin are detected not only in most of the epithelial-origin ovarian tumors but also in the serum samples collected from the epithelial ovarian cancer patients. High levels of Activin are thought to be responsible for promoting disease progression and are predictive of worst disease prognosis for ovarian cancer patients [10]. FST binds to Activin in an antagonistic manner and elevated expression of cellular FST may leads to cytoprotection role in ovarian cancer patients. A recent study has demonstrated that transient transfection with wild-type (wt) *FST* was shown to inhibit metastasis in small-cell lung cancer cell lines [11]. In contrast, significantly higher (P<0.05) concentrations of FST have been reported in ovarian cancer patients when compared with age-matched healthy volunteers.

Members of the TGF-ß superfamily have been shown to modulate the growth of normal human ovarian epithelial cells *in vitro* [12], [13]. Thus, the mutations in TGF-ß receptors or their intracellular signaling molecules such as SMAD proteins have been implicated in the development of several cancers [14], [15], [16], [17], [18] including human ovarian cancer [19], [20], [21]. Another member of the TGF-ß superfamily, inhibin, is a functional antagonist of activin and has been shown to modulate growth of normal human ovarian epithelial [22], [23]. Inhibins, produced by human gonads, play crucial role in attenuating Activin-regulated signaling in human gonodal system [22]. Inhibin is activated by the presence of a co-factor, beta-glycan, which in turn competes with activin to bind with the activin receptors (Atria and ActRIIB), thus antagonizing Activin-regulated signaling cascade [24]. Furthermore, total inhibin has been suggested as a potential serum marker for epithelial-origin human ovarian cancer. Additionally, follistatin related gene protein (FLRG), which shows a strong homology with FST, and has been shown to interact and block the function of the TGF-ß superfamily ligands including Activin, Bone morphogenetic protein (BMP)-2, BMP-6, BMP-7, and GDF-8. FLRG is primarily expressed in heart, lung, kidney, and placenta and can be stimulated by TGF-ß, Activin and SMAD proteins [25]. FLRG expression is reported to be highly variable in several cancer lines and tissues. One recent study showed that down-regulation of FLRG inhibits human breast tumor growth [26].

Several studies have proposed that the targeted therapeutic strategies, specifically molecular mediators that down-regulate endogenous Activin expression levels may slow down or inhibit cancer progression [27]. Thus, a possible role of FST as an Activin antagonist in the pathogenesis of ovarian cancer requires further investigation. In this study, we used immortalized ovarian surface epithelium (IOSE) cells from normal human ovary (IOSE 7576 and IOSE 397) and from an ovarian cancer patient with *BRCA1* mutation, IOSE 592F, to investigate the role of BRCA1 in mediating FST secretion in these cells. We also constructed several stable BRCA1 clones in SKOV3 ovarian adenocarcinoma cell line that ectopically express BRCA1 protein (*i.e.* BRCA1-SKOV3). We initially performed microarray analysis using BRCA1-SKOV3 clone and control neo clone to identify early biomarkers in ovarian cancers. Next, we validated our results using real time-PCR analysis and found that *FST* and *SMAD6* were up-regulated in BRCA1-SKOV3 clone #19 as well as in all of the IOSE cell lines. Taken together, these results suggest that the loss or diminished levels of FST secretion in ovarian cells may potentially serve as a marker for human ovarian carcinogenesis.

## Methods

### Expression vectors and reagents

The wild-type (wt) BRCA1 expression plasmid was created by cloning BRCA1 cDNA into the pcDNA3 vector (Invitrogen, Carlsbad, CA) using artificially engineered 5′ *Hind*III and 3′ *Not*I sites [28]. Dimethylsulfoxide (DMSO), ß-mercaptoethanol, and all other chemicals were obtained from Sigma (St Louis, MO) unless otherwise stated.

### Cell lines and culture conditions

SKOV3 cell line was obtained from the American Type Culture Collection (Manassas, VA). The cells were cultured in Dulbecco's modified Eagle's medium (DMEM) supplemented with 10% fetal bovine serum (FCS), non-essential amino acids (100 mM), L-glutamine (5 mM), streptomycin (100 mg/ml) and penicillin (100 U/ml) (all from Lonza, Inc., Walkersville, MD). The cells were maintained at 37°C in a humidified atmosphere of 95% air and 5% CO_2_ and sub-cultured twice weekly, using trypsin (Lonza) [29]. Stable BRCA1-SKOV3 clones, as well as empty-vector transfected (neo) stable clones were generated via transfection with the relevant vectors followed by selection with Geneticin (Invitrogen).

### Immortalized Ovarian Surface Epithelium (IOSE) cells

The immortalized cell lines (IOSE 7576, IOSE 397 and IOSE 592F) were kind gift from Dr.Nelly Auersperg of the Canadian Ovarian Tissue Bank (University of British Columbia, Vancouver, Canada). Briefly, ovarian surface epithelial cells were scraped from human ovarian surface tissue and cultured in 1∶1 mixture of medium 199 (Invitrogen) and MCDB 105 (Sigma) supplemented with 10% FBS, NaHCO3 (2.2 g/L), L-Glutamine (5 mM), penicillin (100 U/ml) and streptomycin (100 µg/ml) (all from Invitrogen). The low-passage cultures of isolated human ovarian surface epithelium cells were then immortalized by transfecting with SV40 large-T antigen viral particles [30]. The morphological characteristics and the growth pattern of IOSE cell lines were seen to mimic ovarian surface epithelial cells in the extracellular matrices. Further, IOSE cells exhibit strong morphological resemblance with the early neoplastic ovarian cells in humans and thus serve as an excellent model for the *in vitro* studies focused on human ovarian carcinogenesis [30], [31].

### Affymetrix Oligonucleotide Microarrays

Affymetrix microarray analyses were performed at Georgetown University's Lombardi Cancer Center Genomics and Epigenomics Shared Resources core facility. RNA isolation, cDNA synthesis, gene chip hybridizations, and data analysis were performed as described earlier [32]. The empty vector and the stable BRCA1 clone in SKOV3 cells that were used to generate microarray data thoroughly described in our earlier manuscript [32]. The gene chips used for these experiments were Human Genome U133 Plus 2.0 Array. All microarray data was MIAME (Minimum Information About a Microarray Experiment) compliant and the raw data has been deposited in Gene Expression Omnibus database (GEO) as describe in our earlier manuscript [32]. The accession number for this submission is GSE30296. Hierarchical clustering of the selected genes was done as before [32].

### Transient transfections

Proliferating SKOV3 cells were transfected overnight with the indicated expression vectors (4–6 µg of plasmid DNA per well of a 6-well plate or 20–25 µg per 100 mm dish) using Lipofectamine 2000 (Invitrogen) as described earlier [29], [33]. Next day, cells were washed with 1X PBS followed by incubation with fresh culture medium for up to 24–48 hr for gene expression.

### Small interfering (si) RNA treatments

Asynchronously proliferating cells were pre-treated with gene-specific siRNA (100 nM for 72 hr) using siPORT Amine (Ambion, Foster City, CA). The efficiency of knock-down was confirmed via immunoblotting for the target protein(s). Following siRNAs were used in this study: control siRNA [ON-TARGET *plus* Non-targeting siRNA (Cat# D-001810-01, Dharmacon, Chicago, IL)]; FST specific siRNA was obtained from Santa Cruz Biotechnology, Santa Cruz, CA; BRCA1-specific siRNA [pool of two custom synthesized siRNA from Dharmacon (sequences 5′→3′ CAGCTACCCTTCCATCATA and CTAGAAATCTGTTGCTATG)]. Both FST and BRCA1 siRNA were targeted against the ORF of the respective genes.

### Semi-quantitative Reverse Transcriptase – Polymerase Chain Reaction (RT-PCR) analysis

Semi-quantitative RT-PCR assays were performed as described before [29]. Briefly, cells were treated as indicated above and total RNA was extracted using the RNase Easy Mini Kit (Qiagen, Valencia, CA). Aliquots of total RNA (50 ng) were reverse transcribed using 50 units of Superscript III reverse transcriptase (Invitrogen) in a reaction volume of 20 µl. Semi-quantitative PCR amplification was performed using 1 µl aliquot from each sample of the transcribed cDNA, using hot-start Taq polymerase (Denville, South Plainfield, NJ). DNA was first denatured for 5 min at 95°C, and then amplified using cycles of 30 sec at 95°C, 30 sec at the specific annealing temperature, and 1 min at 72°C, with final 10 min incubation at 72°C. The cycle number was adjusted so that all reactions were within the linear range of PCR product amplification. The forward and reverse primers used, annealing temperatures, and expected sizes of the PCR products are shown in Table 1. Quantitative mean analysis from at least three independent experiments is represented along with standard error of measurements (± SEMs).

**Table 1:** Primers, annealing temperatures, and product sizes for PCR amplification.

Gene	Direction	Primer sequence (5′→3′)	Annealing Temperature	Size (bp)
BRCA1	Forward	AAC CCC TTA CCT GGA ATC TG	57°C	225
	Reverse	TCC CTG CTC AGA CTT TCT TC		
FST	Forward	CGG ATC TTG CAA CTG AAT CT	58°C	203
	Reverse	TCA AAG CCC TCT GAT ACA GC		
SMAD6	Forward	AAG AGAAAC TCG CTC CAA GT	56°C	177
	Reverse	GAA AGG CAG GCT TGT TGA TA		
ACVR2B	Forward	CAG CAG ATG TGT CTT TCA CG	58°C	201
	Reverse	CCT CAA TTC CTG GTT ACC T		
Actin	Forward	TAT CGA GCA CGG CAT CAT CA	58°C	288
	Reverse	TAA ATG GGC ACG TTG TGG GT		

### Quantitative real-time-PCR (Q-PCR) analysis

The Q-PCR was conducted by the ABI PRISM 7900HT Sequence Detection System (Applied Biosystems, Foster City, CA) using SYBR Green PCR Master Mix reagent (Applied Biosystems) in a 25 μl reaction volume. 10 ng of cDNA samples with appropriate gene-specific primers were used for each PCR analysis. Cycle threshold values were automatically adjusted, and the fold changes for each pair of gene-specific primers were determined. Q-PCR primers are listed in Table 1 and were obtained from Real Time Primers, LLC., Elkin Parks, PA.

### Western blotting

Cells were treated as indicated and whole cell lysates were prepared as described previously [34]. Briefly, post-treatment cells were washed with 1X PBS and lysed using radioimmunoprecipitation assay (RIPA) buffer (ROCHE, Indianapolis, IN) supplemented with complete Mini, EDTA-free protease inhibitor cocktail tablet (Roche) on ice for 10 min. The lysed cells were rocked at 4°C for 20 min, followed by centrifugation at 12,000×g for 20 min. Aliquots of protein (50 µg) in 4X LDS (lithium dodecyl sulfate) sample buffer (Invitrogen) were analyzed on the pre-cast 4–12% Bis-Tris (BT) gels (Invitrogen). The fractionated proteins were then transferred to polyvinylidene fluoride (PVDF) membranes (Millipore, Billerica, MA) and blocked for an hr in 5% non-fat milk in 1X PBS-Tween 20. Membranes were then incubated with indicated primary antibodies, and subsequently with species-specific horseradish peroxidase (HRP)-conjugated secondary antibody (Santa Cruz). Blotted proteins were visualized using the enhanced chemiluminescence detection system (Santa Cruz). The primary antibodies used were: BRCA1 (C-20, Santa Cruz, 1∶200); FST (K-19, Santa Cruz, 1∶100); Actin (C-11, Santa Cruz, 1∶400), and Activin (ab89307, Abcam, Cambridge MA, 1∶1000).

### Follistatin Assay

Quantitative detection of cellular FST levels was performed using Quantikine Human FST Immunoassay kit (R & D Systems Inc., Minneapolis, MN) that employs the quantitative sandwich enzyme immunoassay (ELISA) method. Briefly, a standard curve was made to quantitate the amount of FST in the culture medium and cell lysates using known FST standards provided in the kit. Cells were treated as indicated, and the medium from the treated cells was diluted 10 times for the assay. Both standards and diluted samples were pipetted into the wells pre-coated with FST monoclonal antibody. The immobilized antibody was allowed to bind to FST present in the samples, followed by the incubation of an enzyme-linked monoclonal antibody specific for FST in each well. A substrate solution was added to the wells following incubation leading to a color development in proportion to the amount of FST bound during the initial reaction step. The intensity of the color is measured at dual wavelength (450–540 nm). The amount of FST in each indicated samples represented as means ± SEMs.

### Cell migration Assay

Cell migration was assayed using CytoSelect 96-well Cell Migration Kit, 8 µM, Fluorometric (Cell Biolabs, Inc. San Diego, CA) as per manufacturer's instructions, which is based on Boyden Chamber method. Briefly, treated/untreated cells were suspended in a serum free culture medium and then plated in the upper membrane chamber of the cell migration plate. The membrane chamber containing cells were then placed on the top of the feeder tray to facilitate cell migration across the membrane. The chemo-attractant used in the feeder tray was complete cell culture medium containing 10% FCS. The complete assembly was incubated at 37°C cell culture incubator for 24 hours. The migrated cells from each sample was collected from the feeder tray; lysed using a lysis buffer consisting of fluorescent CyQuannt GR dye, and the total fluorescence of the migrated cells was quantified using a Victor 1420 fluorescence microplate reader (Perkin-Elmer Wallac, Inc., Waltham, MA) at dual wavelength (480–520 nm). The observed fluorescence units (FU) are directly proportional to the number of cells migrated; higher the observed fluorescence higher the cell migration. A standard curve was constructed for each cell line using an incremental cell density as per manufacturer's instructions, and the data obtained from the standard curve from each cell line (treated/untreated) are represented as means ± SEMs.

### Cell proliferation Assay

FITC BrdU flow kit from BD Pharmingen (San Jose, CA) was used to perform the cell proliferation assays with the IOSE cell lines as per manufacturer's instructions. Briefly, 10 µM of BrdU was added to asynchronously growing cells in logarithmic phase for an hr to pulse the cells. Cells were then fixed and permeabilized using BD cytofix/Cytoperm buffer (provided in the kit), and subsequently treated with DNase to expose BrdU. FITC antibody was then used to detect BrdU, and then the cells were subjected to a flow cytometer FACSort (Becton Dickinson, San Jose, CA) and the parameters were set according to the manufacturer's instructions. FACS analysis was done in the Flow Cytometry and Cell Sorting Shared Resources at Lombardi Cancer Center, Georgetown University. Data obtained were represented as means ± SEMs of the percent BrdU labeled cells.

### Statistical analysis

The data was statistically analyzed by ‘One way analysis of variance (ANOVA) via Tukey Test’, which is a pair-wise comparison of the mean responses to the different treatment groups using SigmaStat statistical software (Ver 3 for windows). A value of P<0.05 or less was considered significant for all experiments.

## Results

### Stable expression of BRCA1 in SKOV3 cell line

**Figure 1:**
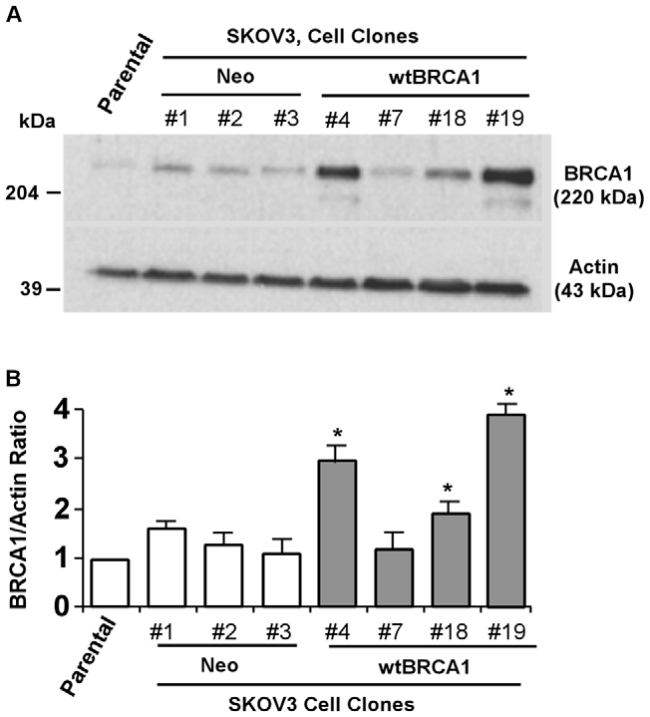
Generation of SKOV3 cell clones. (A) Several stable lines were created using overexpression of BRCA1 and its background vector, pcDNA3 in SKOV3 ovarian carcinoma cells. Expression levels of BRCA1 are shown in all cell clones. Parental indicates non-transfected SKOV3 cells only. Densitometric analysis from three independent immunoblots is given in (B). Error bars are SEMs. * represents P<0.05 (compared to parental).

Unlike breast cancer, there are no well-established guidelines linking *BRCA1* mutations and individual's predisposition to developing ovarian cancer. Here, we utilized human ovarian cancer cell line (SKOV3) as a parent ovarian cancer cell line and generated several stable clones that ectopically expressed wtBRCA1 (BRCA1-SKOV3) and its empty background vector, pcDNA3 (Neo). As seen in Figure 1A, #4, #18, and #19 clones of BRCA1-SKOV3 demonstrated a significant increase in the expression of BRCA1 (P<0.05) compared to parental SKOV3 cells as well as corresponding Neo clones. The densitometric analysis from three independent western blots is shown in Figure 1B as means ± standard error of measurements (SEMs). Thus, generation of SKOV3 sub-cell lines with stable BRCA1 expression would further assist in understanding the effect of BRCA1 in human ovarian carcinoma.

### Differential gene expression due to BRCA1 overexpression in SKOV3 cells

**Figure 2:**
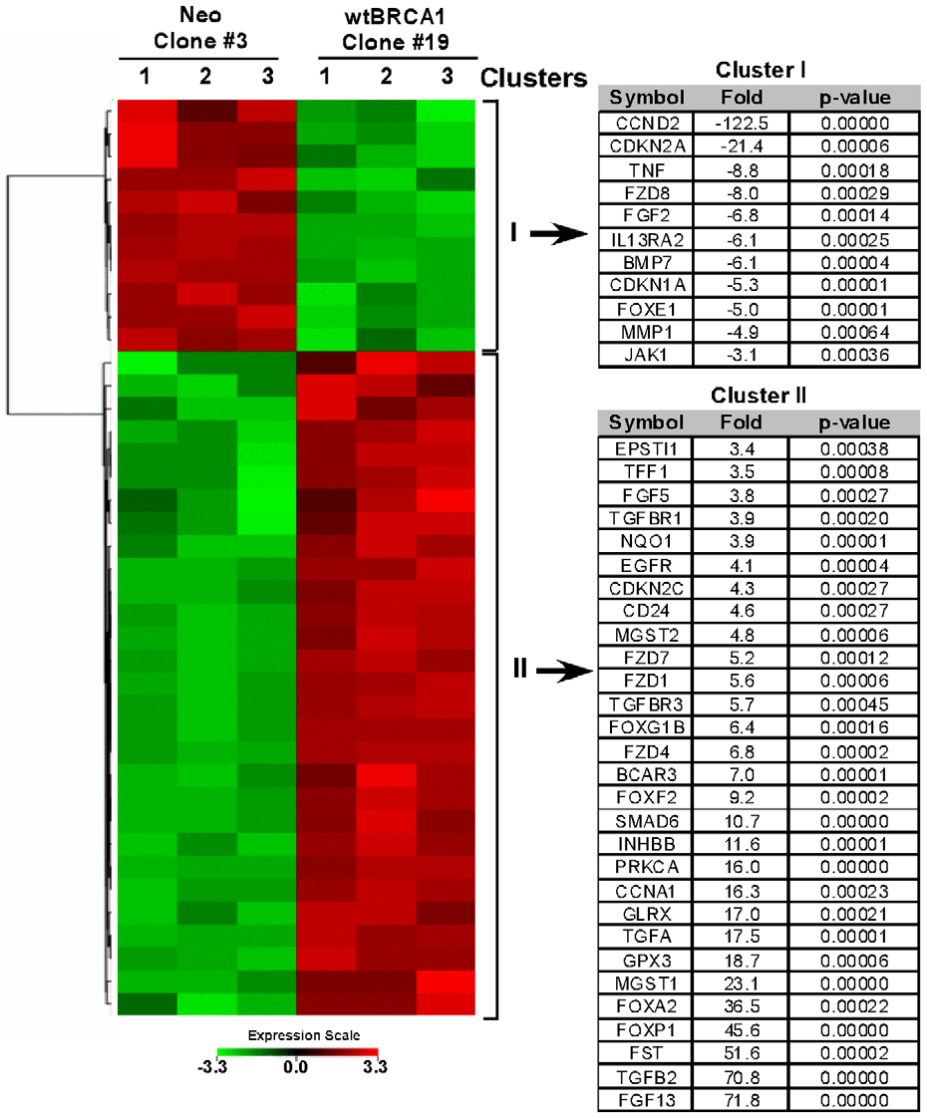
Hierarchical gene clustering generated by the Ingenuity Pathway Knowledge Base. A heat map showing significant changes in a group of selected genes due to overexpression of wtBRCA1 in SKOV3 ovarian carcinoma cells. Three independent microarray analyses were performed. Two clusters were detected, one due to down-regulation of the genes (green) and other due to up-regulation of the genes (red). Fold change and p values of the genes in the clusters are given on the right.

Based on the BRCA1 expression of #19 clone of BRCA1-SKOV3 (hereafter referred as BRCA1-SKOV3 clone #19) was selected and paired with #3 of Neo clone (hereafter referred as Neo clone #3) for the Affymetrix microarray analysis. RNA from three separate culture passages of BRCA1-SKOV3 clone #19 cells and Neo clone #3 cells was isolated and subjected to the microarray analysis (see methods for details). Next, we constructed a hierarchical clustering of gene expression that were significantly (P<0.05) altered in the BRCA1-SKOV3 clone #19 cells and compared with the Neo clone #3 cells. We observed over 10 fold up-regulation in 274 genes and more than 4 fold down-regulation in 279 genes for BRCA1-SKOV3 clone #19 cells when compared with Neo clone #3 (see Table S1 as well as corresponding heat map in Figure S1). Using this microarray data, Ingenuity Pathway analysis revealed that BRCA1 overexpression in SKOV3 cells (BRCA1-SKOV3 clone #19) modulates several critically important signaling and metabolic pathways (see Table S2 for the up-regulated genes and Table S3 for the down-regulated genes in BRCA1-SKOV3 clone #19). The list of genes was further narrowed down to selected 40 genes based on about 3 fold-change in the expression levels when compared to control, and with significant p values (P<0.05). The selected groups of genes were found to be mostly responsible for cell growth, cell cycle progression and oxidative stress response, which were of primary interest for the current study. Hierarchical clustering was redone using these selected 40 genes dataset for up-regulated (red) or down-regulated (green) genes due to wtBRCA1 overexpression in BRCA1-SKOV3 cell clone # 19 (Figure 2). The resultant two clusters of genes along with their fold-change and p value are provided in the right panel of Figure 2.

FST, an antagonist of Activin, is responsible for early ovarian development; however, not much is known about the cellular regulation of FST in human ovarian tissue. Elevated FST level in human ovarian cells are suggested to be cytoprotective against ovarian carcinogenesis. We have observed 51.6 fold higher expression of FST in BRCA1-SKOV3 clone #19 when compared with the Neo clone #3 cells. Inhibin was also found to be significantly (P<0.05) up-regulated (11.6 fold) in BRCA1-SKOV3 cells. Several studies have suggested the potential role of inhibin as a biomarker for ovarian cancer of epithelial origin [35]. A few studies proposing inhibin as a serum marker in ovarian carcinogenesis revealed that inhibin levels are elevated in approximately 70–80% of the mucinous type ovarian cancer patients, and in 15–35% of non-mucinous type epithelial ovarian cancer cases [36], [37]. Studies have further shown that in the post-menopausal women, total inhibin can be combined with CA-125 for the efficient diagnosis of ovarian epithelial carcinogenesis [35].

Additionally, we found that several other members of TGF-β family such as TGF-ß2, TGF-ßR1, TGF-ßR3 and an inhibitory SMAD, SMAD6, were also up-regulated in BRCA1-SKOV3 clone #19 (Figure 2). Thus, the hierarchical clustering approach was not only able to confirm number of previously known potential markers in ovarian cancer but also reveal several other putative molecular targets that may be of interest for designing targeted treatment for human ovarian cancer.

### Validation of Affymetrix microarray results by semi-quantitative RT-PCR

**Figure 3:**
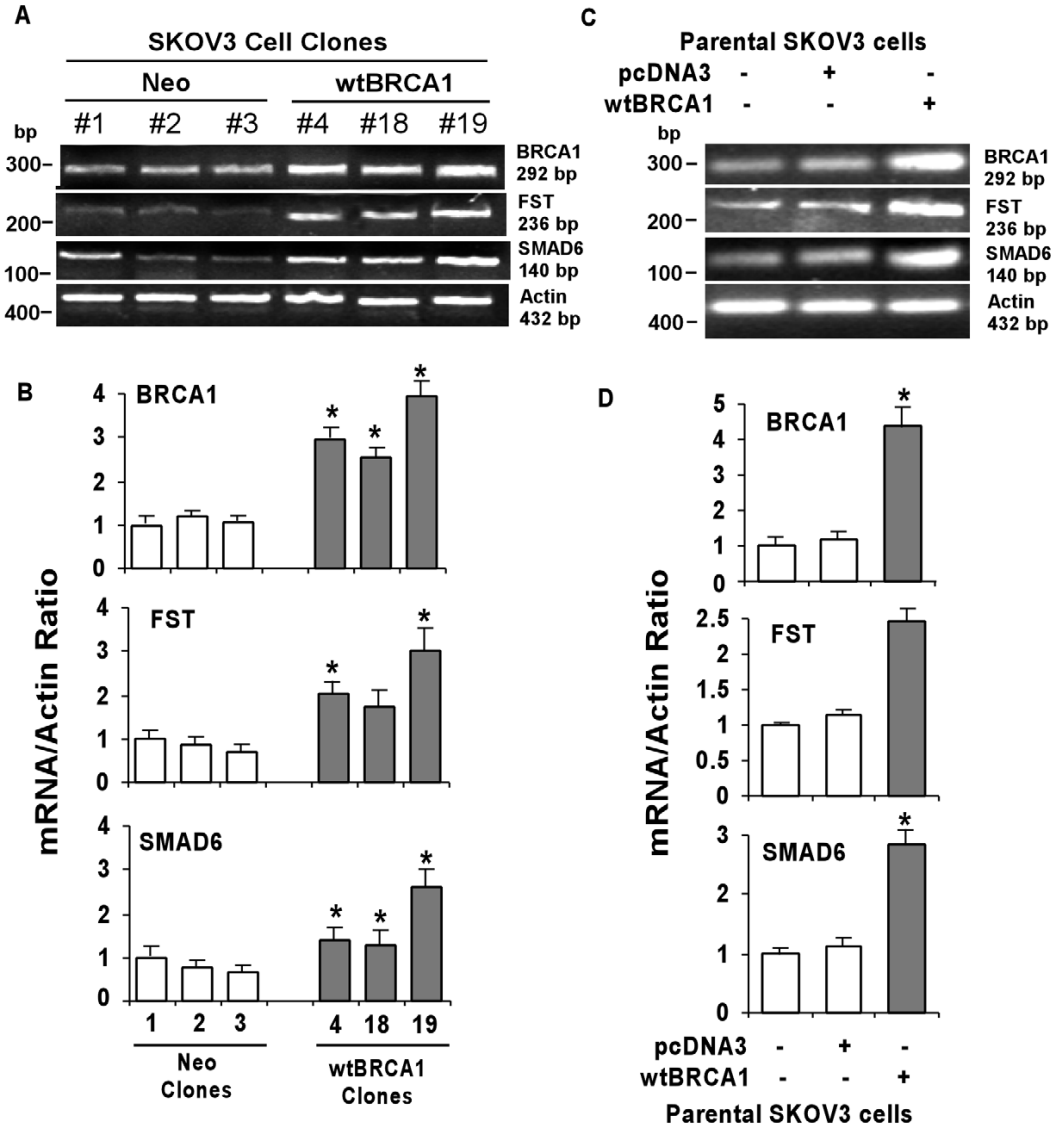
BRCA1 regulates the mRNA expression of FST. (A) Proliferating stable clones of both Neo (pcDNA3-SKOV3) and wtBRCA1 (BRCA1-SKOV3) were harvested for semi-quantitative RT-PCR assay as described under ‘Methods’. Bands corresponding to the respective genes are indicated on the right. (B) The PCR bands from three independent experiments of each indicated genes were quantified by densitometry and mRNA levels were normalized to Actin. (C) Proliferating SKOV3 cells were transiently transfected either with wtBRCA1 or background pcDNA3 vector and the isolated RNAs was assayed by semi-quantitative RT-PCR. The results were quantified as above and represented by bar diagram (D). Error bars are SEMs. *P<0.05 (relative to each controls).

We have used RT-PCR to validate the results obtained from the microarray analysis. Three individual stable clones for each Neo and BRCA1-SKOV3 cell lines were used to perform semi-quantitative RT-PCR. We found a significant correlation (P<0.05) between the microarray and RT-PCR results. Figure 3A shows representative mRNA expressions of the indicated genes in all groups of cells. Figure 3B demonstrates quantitative estimation of the mRNA expressions of all the genes under investigation based on at least three independent experiments. In all cases, mRNA expression of FST was significantly (P<0.05) higher in BRCA1-SKOV3 clones when compared to Neo clones, validating the results observed in microarray analysis, although it is necessary to note that the degree of fold-change is variable; possibly due to difference in the sensitivity of these two techniques. BRCA1-SKOV3 clone #19 demonstrated maximum up-regulation of BRCA1 among the stable cell lines; hence all the future experiments were performed using this clone, as shown above, and was compared with Neo clone #3. In addition, RT-PCR also confirmed the up-regulation of SMAD6 in the BRCA1-SKOV3 clone #19 (Figure 3A). Furthermore, we investigated the effect of transient expression of BRCA1 in the parental SKOV3 cells. The results shown in Figure 3C are consistent with the results observed in Figure 3A and 3B. FST mRNA expression was found to be significantly (P<0.05) elevated in BRCA1 overexpressed SKOV3 cells when compared to the background vector transfected (pcDNA3) as well as parental SKOV3 cells. In addition, mRNA expressions of SMAD6 were also elevated in the wtBRCA1 overexpressing cells (Figure 3C). Densitometry of the expression of the genes of interest from at least three independent experiments are given in Figure 3D.

### BRCA1 regulates FST secretion in SKOV3 ovarian carcinoma cells

**Figure 4:**
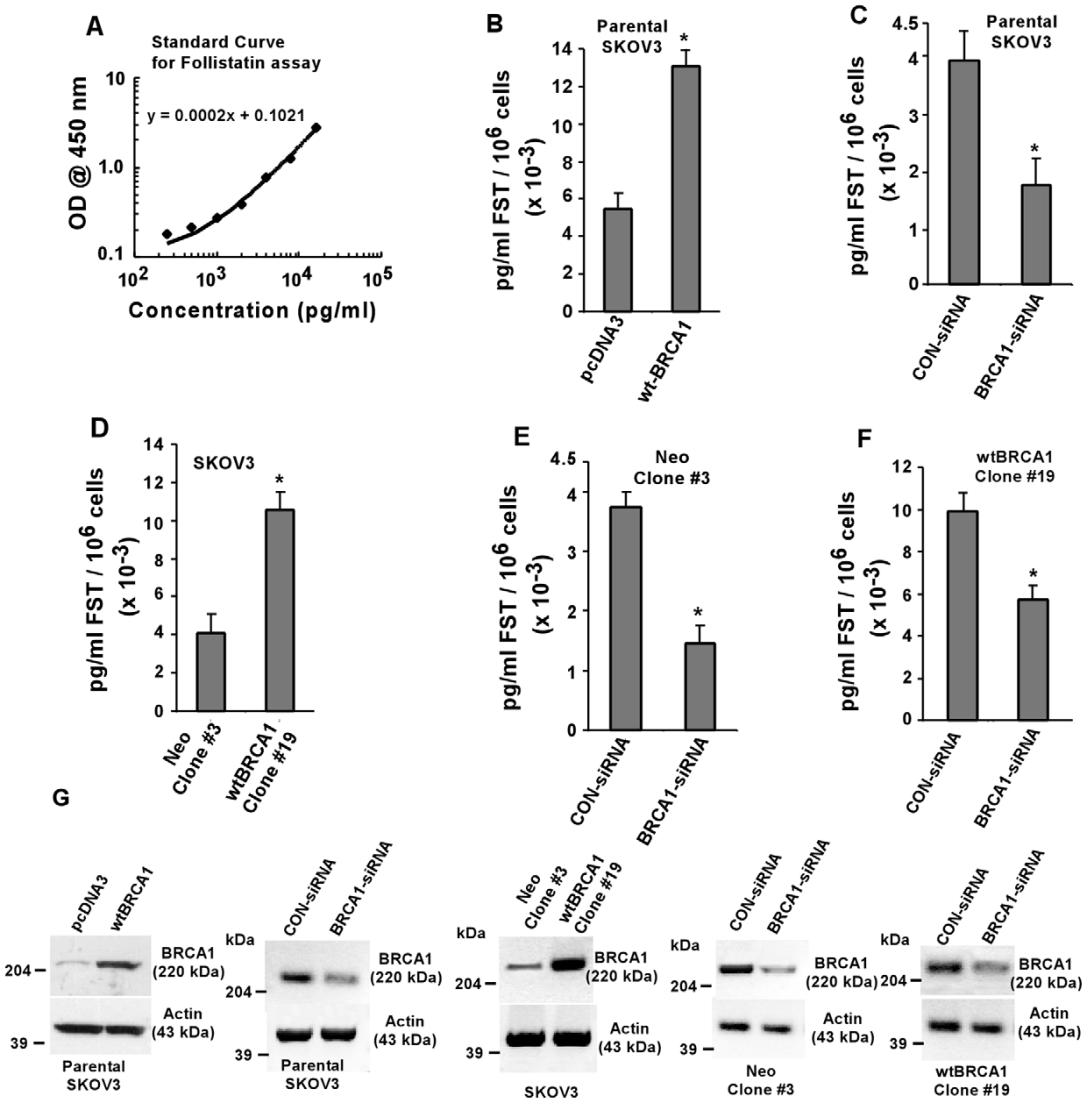
Effect of ectopic expression of BRCA1 on FST secretion. (A) Standard curve was generated with purified human FST as indicated in the ‘Methods’, which was used to quantitate unknown FST concentrations in the indicated samples. SKOV3 cells were transfected as before, either for BRCA1 overexpression (B) or for BRCA1 underexpression (C), and FST assays (see methods) were performed with the diluted culture medium. The same FST assay was performed with the stable clones as indicated (D) as well as with the stable cells where expression of BRCA1 was knocked down by BRCA1-siRNA (E–F). Error bars are SEMs. *P<0.05 (relative to each control). Expression of BRCA1 in the above samples is given in (G).

FST is secreted by ovarian follicular fluid in the extracellular region, and is essential for early ovarian development [38]. *In vitro* experiment with cultured ovarian cells exhibit differential levels of FST secretion in the culture medium depending upon cell treatments and culture conditions. Thus, a diluted medium serves a good source to measure the secretion and expression of FST in a particular cell type. Figure 4A demonstrates a standard curve obtained with the purified FST protein, which was used to quantitate the amount of secreted FST in the samples under investigation. Our results demonstrate that BRCA1 expression in SKOV3 cells shows approximately 2 fold higher secreted FST when compared to pCDNA3-transfected cells (Figure 4B). Additionally, we performed similar quantitative FST assay using SKOV3 cells where-in BRCA1 was knocked-down by BRCA1-specific siRNA. Figure 4C showed at least 50% decrease in FST expression in BRCA1 knock-down cells when compared to cells treated with control siRNA. Next, we performed the same FST assay with the stable BRCA1 cell clones in SKOV3 cells as described earlier. BRCA1-SKOV3 clone #19 cells showed increased FST secretion than neo clone #3, as expected (Figure 4D). Moreover, we also investigated the secreted levels of FST in the stable lines following down-regulation of BRCA1 in the same. Down-regulation of BRCA1 in either Neo clone #3 (Figure 4E) or BRCA1-SKOV3 clone #19 (Figure 4F) significantly (P<0.05) inhibited the secretion of FST in cell culture medium compared to their respective controls. Protein expression levels of BRCA1 in all of the above samples are shown in Figure 4G. Data obtained from this quantitative FST assay suggests that induction in cellular BRCA1 levels in turn stimulates extracellular FST secretion in human ovarian cancer cells.

### BRCA1 regulates mRNA expression of FST in human immortalized ovarian surface epithelial (IOSE) cells

Recent statistics on ovarian cancer published by the National Cancer Institute (NCI) reveal that more than 85% of the ovarian cancers arise within the ovarian surface epithelial (OSE) cells. OSE is a single layer squamous epithelium with a few stromal features that lose its stromal characteristics during neoplastic transformation. Thus, we expanded our investigation with cells derived from human OSE, which were immortalized by SV40 T antigen (IOSE 7576 and IOSE 397). In addition, we also used a unique IOSE cell line, IOSE 592F, which was developed from an ovarian cancer patient harboring mutation in *BRCA1* gene [in exon 11, position 3819del5 (GTAAA)]. BRCA1 expression levels in both IOSE 7576 and IOSE 397 cells were manipulated by using either wtBRCA1 plasmid for overexpression studies or BRCA1-specific siRNA for underexpression studies. These cells were then subjected to quantitative RT-PCR as described before (see methods).

**Figure 5:**
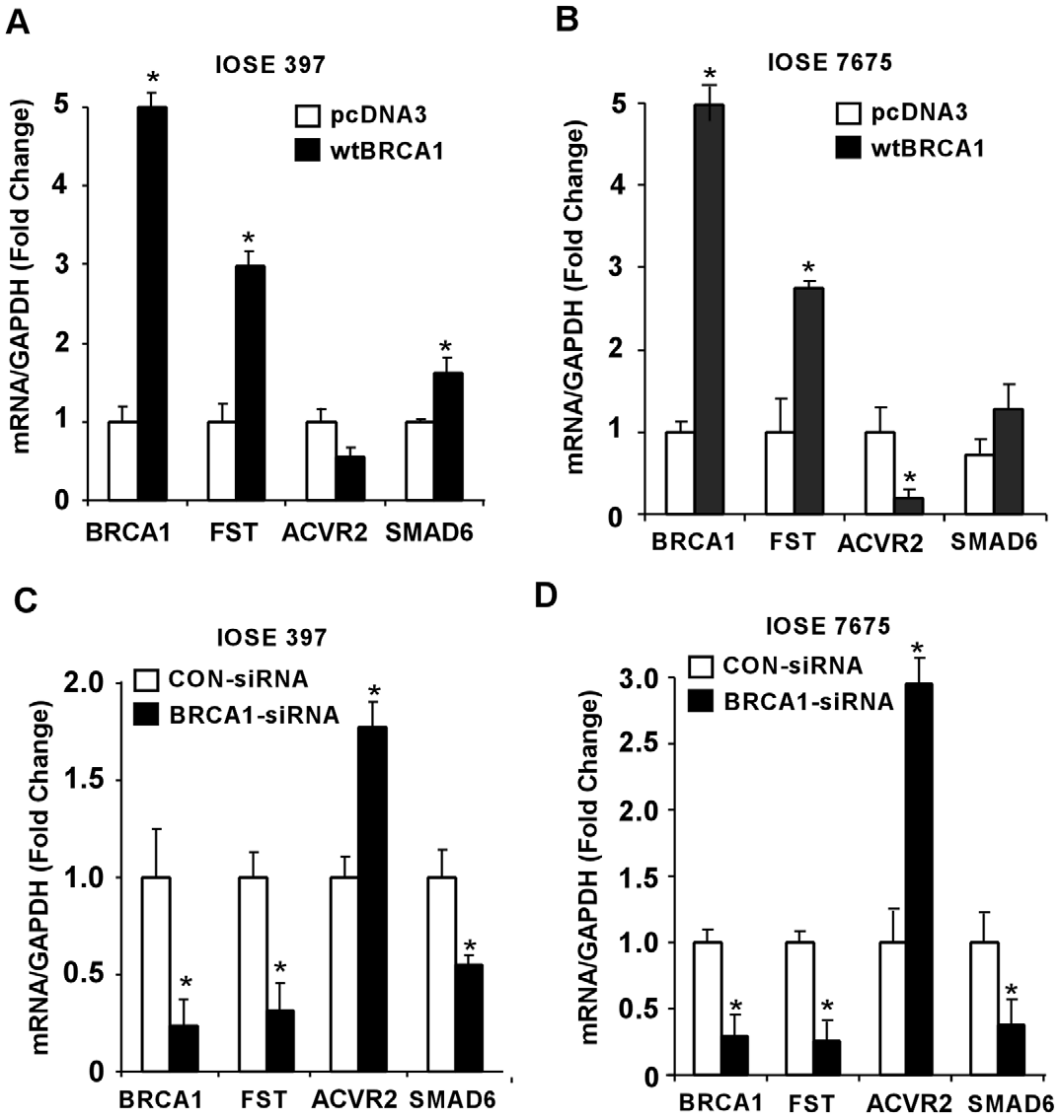
Q-PCR analysis with the immortalized ovarian surface epithelium cells (IOSE). BRCA1 expressions in IOSE cells was modulated either with wtBRCA1 or BRCA1-siRNA along with appropriate the controls. Total RNA was isolated from all the cells and subjected to real time PCR analysis. Overexpression of BRCA1 in IOSE 397 (A) and IOSE 7576 (B) cells induce overexpression of FST and SMAD6 but knock down the expression of ACVR2B. In contrast down-regulation of BRCA1 in IOSE 397 (C) and IOSE 7576 (D) cells, demonstrates the reverse effect. mRNA expression of GAPDH was used for normalization in each case. Error bars are SEMs. * P<0.05 (relative to each control).

IOSE 397 and IOSE 7576 cells that were manipulated to ectopically express wtBRCA1 showed over 2 fold increase in FST mRNA expression when compared to pcDNA3-transfected cells (Figure 5A and 5B). mRNA expression for SMAD6 also showed induction with the up-regulation of BRCA1 mRNA in these IOSE cells (Figure 5A and 5B). Interplay between Activin, Inhibin, and FST were shown to play a part in the development of ovarian carcinogenesis. Elevated levels of Activin were found in ovarian tumor cells that subsequently induce cellular proliferation in the same cells. Conversely, FST act as a regulator by binding to Activin and antagonizing its tumor promoting function, specifically cell proliferation. Thus, high expression of FST may possibly result in low expression of Activin and its receptors. Figure 5A (IOSE 397) and Figure 5B (IOSE 7576) demonstrate that expression of ACVR2B, a receptor for Activin, was knocked down with the up-regulation of BRCA1 in these cells when compared to the pcDNA3-transfected cells. The down-regulation effect of ACVR2B is significant (P<0.05) in case of IOSE 7576 cells.

In complement to the overexpression studies, we underexpressed BRCA1 in both IOSE 7576 and IOSE 397 cells, using BRCA1-specific siRNA. Cellular mRNA levels of FST were down-regulated to 60–70% in both IOSE cell lines where BRCA1 expression was knocked down (Figure 5C and Figure 5D) when compared with the control siRNA transfected cells. On the contrary, mRNA expression of ACVR2B was elevated with the down-regulation of BRCA1 in both IOSE 397 (Figure 5C) and IOSE 7576 (Figure 5D) cells. As expected, the mRNA expression of SMAD6 was decreased upon BRCA1 knock-down in both IOSE cells (Figure 5A–D). Thus, our results from IOSE cell lines as well as BRCA1-SKOV3 cell clone # 19 indicate that changes in the endogenous BRCA1 levels directly affects the mRNA expression pattern of FST in human ovarian cells.

### Differential secretion of FST by human immortalized ovarian surface epithelial (IOSE) cells

**Figure 6:**
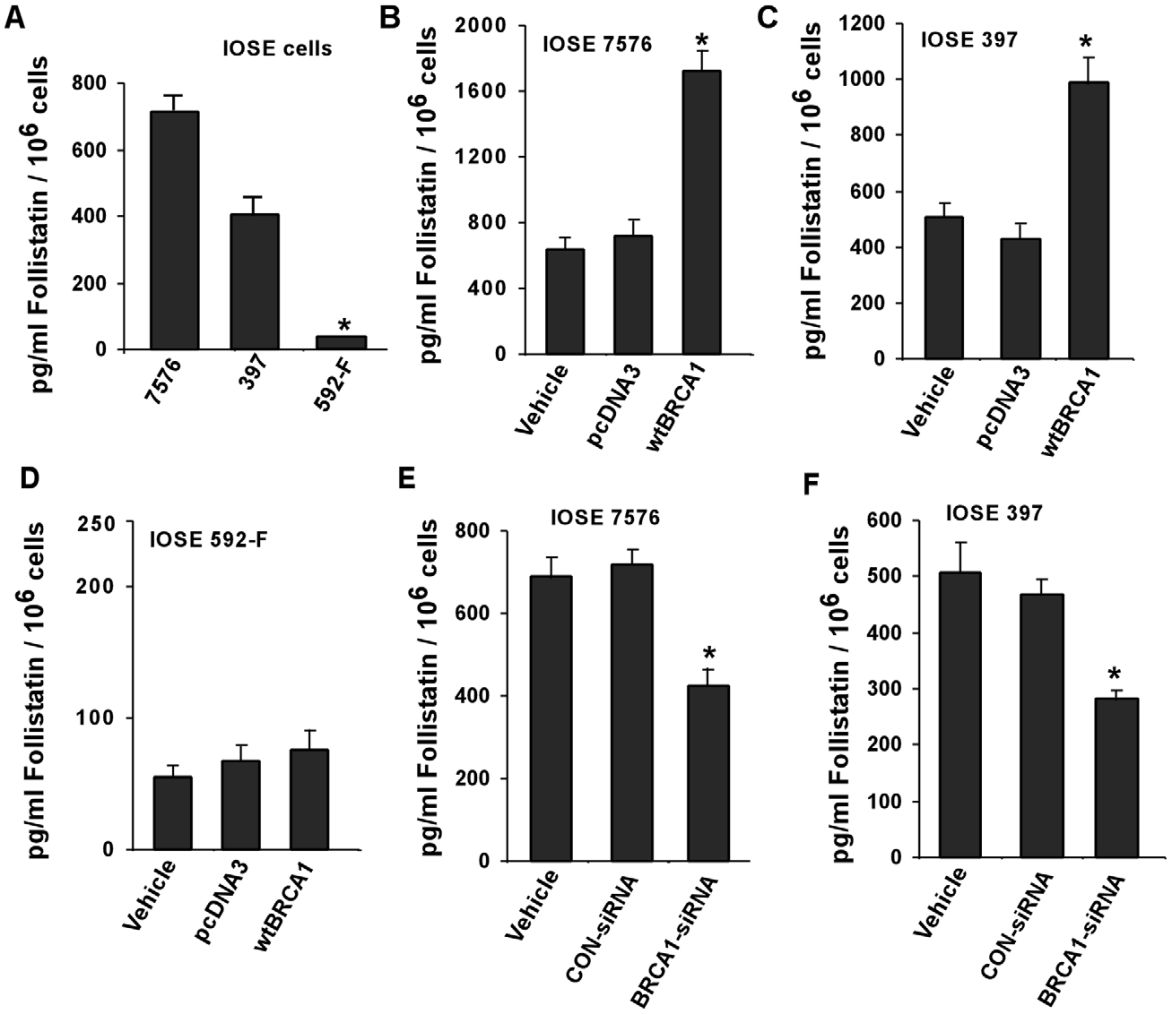
FST assay with IOSE cell line. (A) IOSE 7576, IOSE 397 and IOSE 592F were grown in 10 cm tissue culture plates and the culture medium was subjected to FST assay as described under ‘Methods’. IOSE 7576 cells (B), IOSE 397 cells (C) and IOSE 592F cells (D) were transiently transfected with wtBRCA1 for 48 hr and then FST assays were performed with the culture medium. Additionally, BRCA1 expression was knocked down in both IOSE 7576 (E.) and IOSE 397 (F) cells and then subjected to FST assays. Error bars are SEMs. * P<0.05 (relative to each control).

Subsequently, we demonstrated the effect of FST expression with respect to BRCA1 expression in IOSE cell lines. Figure 6A compares the levels of FST secretion by all of the IOSE cells under investigation. Although both IOSE 7576 and IOSE 397 were derived from normal human ovarian surface epithelium, they show different basal level of FST. These differential levels of FST may be, in part, due to the different genetic background of the individuals that served as a source for isolating these cells. IOSE-592F secreted significantly (P<0.05) low levels of FST in the medium when compared to both normal IOSE cell lines (Figure 6A), consistent with our previous observations. Next, we manipulated the expression of BRCA1 in IOSE cells as before and performed similar FST assay. As seen in Figure 6B and Figure 6C, BRCA1 overexpression in IOSE 7576 and IOSE 397 cells significantly (P<0.05) elevated FST secretion when compared to vehicle and/or pcDNA3-transfected IOSE cells. We further investigated whether overexpression of BRCA1 in IOSE 592F could successfully alter the cellular levels of FST secretion. Surprisingly, induction of BRCA1 levels by wtBRCA1 failed to stimulate the secretion of FST in IOSE 592F cells (Figure 6D). In addition, we investigated the efficiency of FST secretion in both IOSE 7576 and IOSE 397 where BRCA1 has been knocked down by BRCA1-specific siRNA. We found a significant (P<0.05) reduction of FST secretion in BRCA1 down-regulated cells compared to respective controls supporting the earlier observations (Figure 6E and Figure 6F).

**Figure 7:**
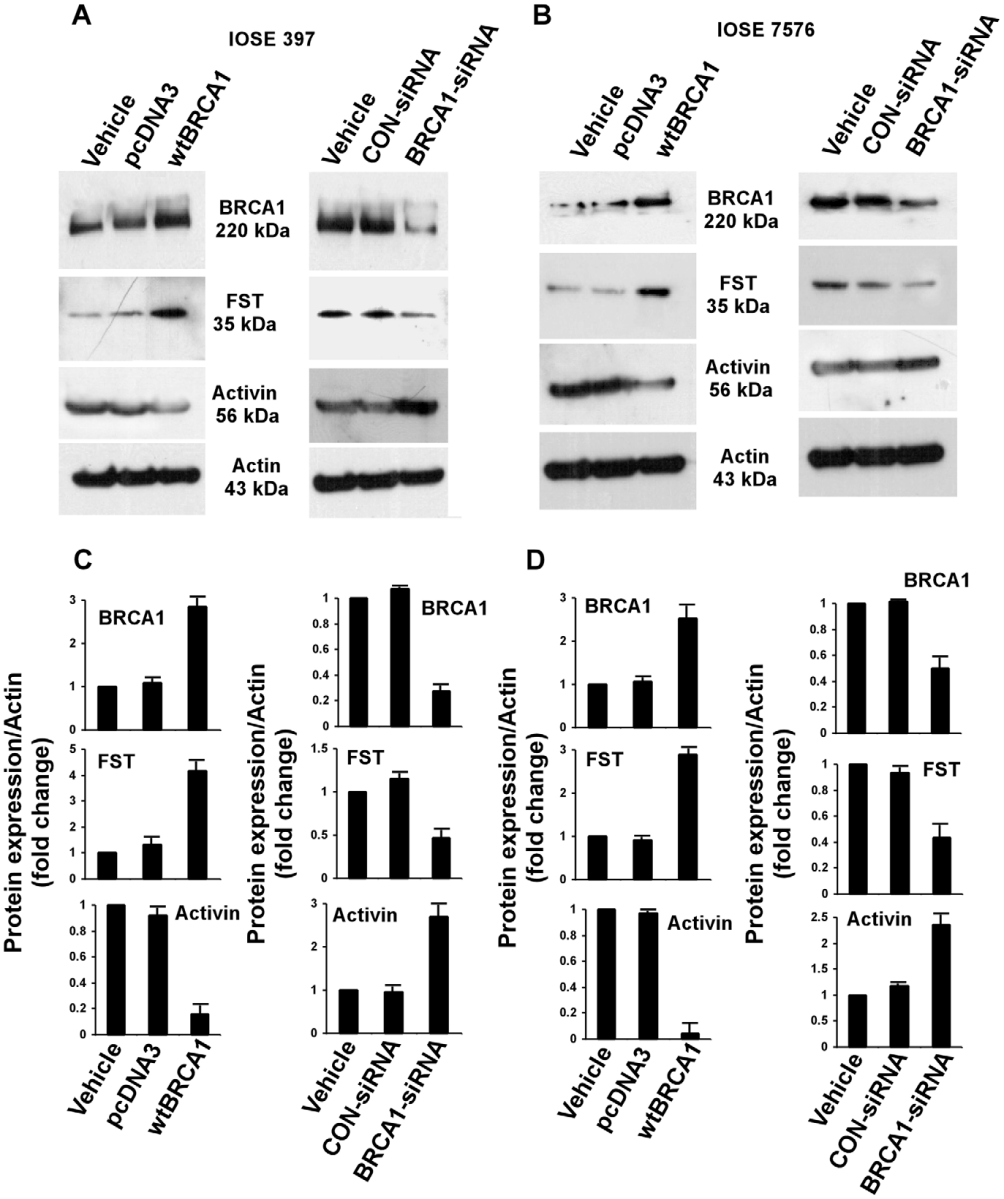
Western blot analysis for IOSE cells. IOSE 397 (A) and IOSE 7576 (B) cells were transiently transfected either with wtBRCA1 or BRCA1-siRNA in each case. Whole cell lysates from the attached cells were fractionated in 4–12% BT gel, and subsequently immunoblotted with the indicated antibodies. Densitometric analyses of the immunoblots shown above are given in C and D respectively.

Furthermore, cells left on the tissue culture plates, after utilizing the culture medium for quantitative FST assay, were subjected to western blot analysis. Cell lysates were fractionated in a 4–12% BT SDS PAGE, and subsequently immunoblotted for BRCA1, FST, Activin and Actin. As expected, IOSE 7576 and IOSE 397 cells overexpressing BRCA1 showed increase in FST expression, whereas knock-down of BRCA1 by BRCA1-siRNA decreased FST expression in these cells (Figure 7A and Figure 7B). In contrast, expression of Activin was down-regulated in BRCA1 overexpressing IOSE 7576 and IOSE 397 cells. On other hand, Activin expression was up-regulated with knock-down of BRCA1 and FST (Figure 7A and Figure 7B). Densitometric analyses based on three independent experiments are shown in Figure 7C and 7D.

### FST regulates cell proliferation and cell motility in human ovarian cells

**Figure 8:**
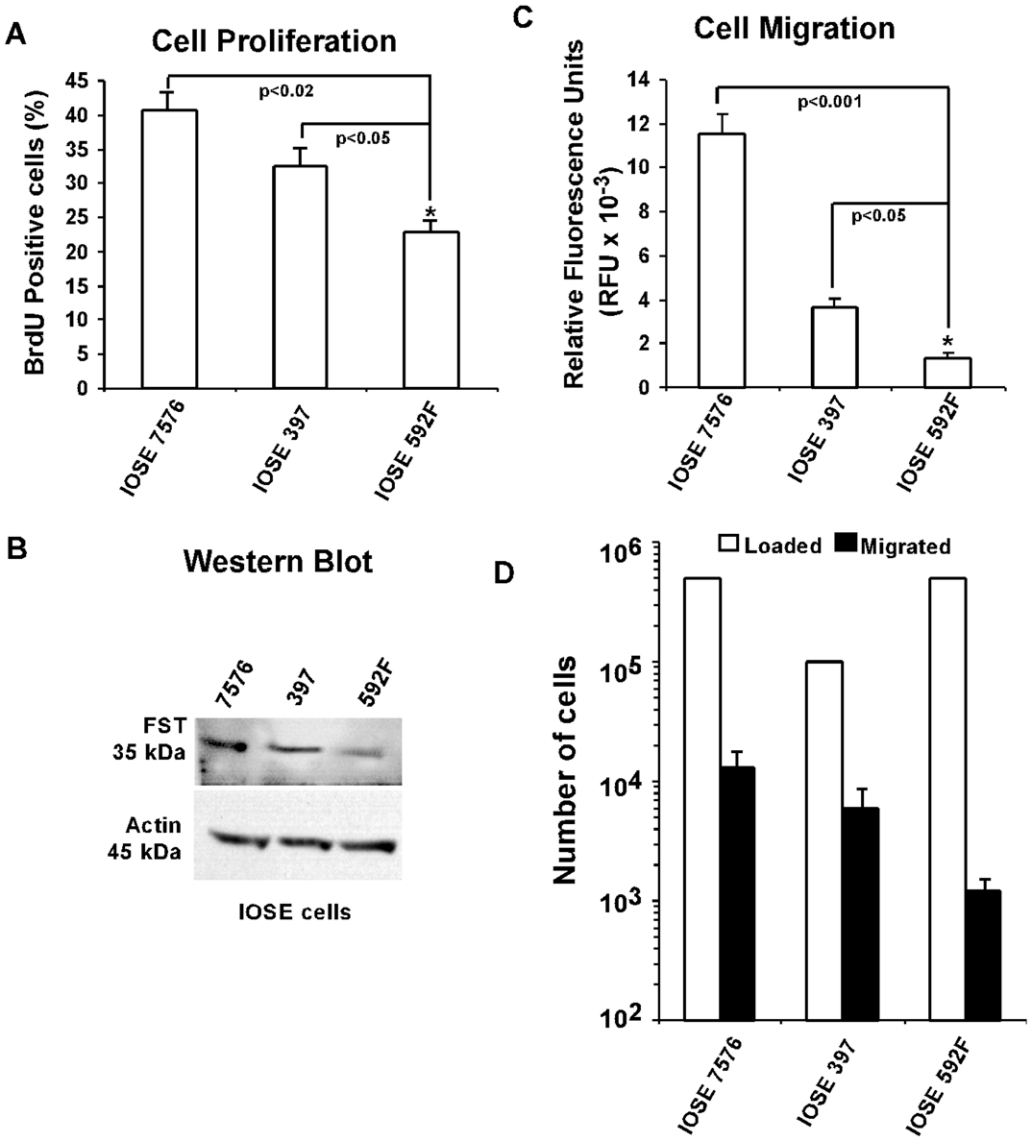
Cell proliferation and cell migration assay with IOSE cells. (A) Comparative analysis of cell proliferation for IOSE 7576, IOSE 397 and IOSE 592F cell lines. (B) Western blot analysis showing FST levels for IOSE 7576, IOSE 397 and IOSE 592F cell lines. (C–D) Comparative cell migration analysis for IOSE 7576, IOSE 397 and IOSE 592F cell lines. Relative fluorescence units (RFU) was measured using all of the migrated cells to the feeder tray in each sample (C), whereas, analysis of the total number cells loaded in the Boyden chamber verses total number of cells migrated towards the chemo attractant (10% FBS) in each case is shown in (D).

We performed two key functional assays namely cell proliferation assay and cell migration assay using SKOV3 cells as well as IOSE cells to demonstrate a potential role of FST in ovarian cancer development. The differential cellular proliferation rates among IOSE 7576, IOSE 397 and IOSE 592F cells, as observed by the differential BrdU incorporation in DNA of actively dividing cells, showed correlation to the differential expression of FST in these cells indicative of FST's role in cell proliferation. We have shown that IOSE-592F, which is an IOSE cell line with a mutation in BRCA1, has reduced FST expression (Figure 6) and its proliferation rate was lowest when compared with other two normal IOSE cell lines (Figure 8A). Further, we also used cell migration assay to assess the physiological importance of FST in all of the immortalized OSEs as well as BRCA1 overexpressing stable clone # 19 in SKOV3 cells. The differential FST expression pattern in IOSE cell lines is shown in Figure 8B, and was detected by Western blot analysis. Migratory potential of the IOSE cell lines is shown in Figure 8C–D. Consistent with cell proliferation results, IOSE 592F cell line showed significantly (P<0.05) lower cell migration when compared with IOSE 7576 and IOSE 397 further suggesting the correlation between cell motility and differential FST status in these cells.

**Figure 9:**
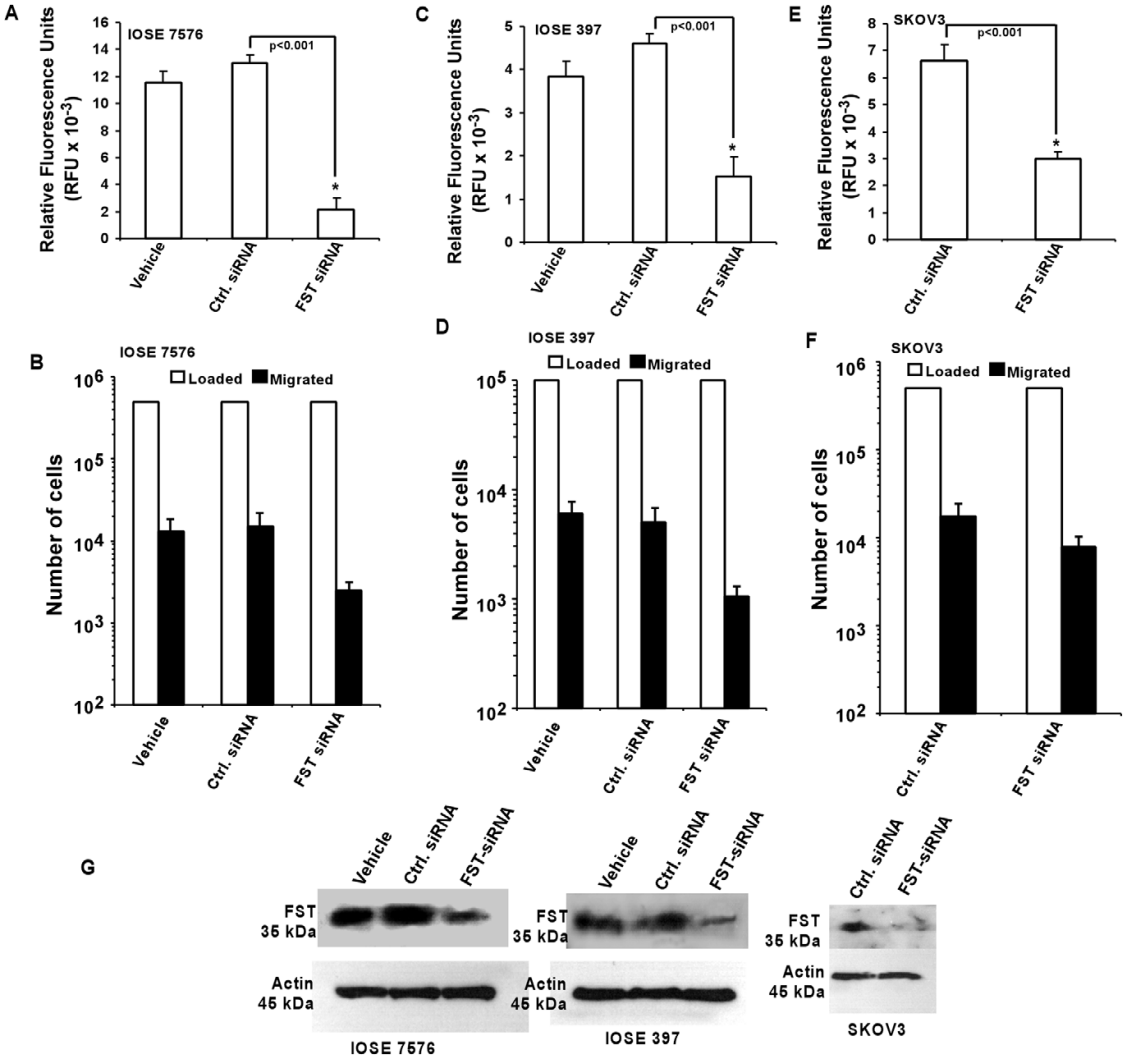
Effect of FST knock down on cell migration. (A–F) Cell migration analysis was performed for IOSE 7576, IOSE 397 and SKOV3 cell lines that were transfected with either control siRNA or FST-siRNA. Relative fluorescence units (RFU) measured from all the migrated cells in each sample are shown in A–C, whereas, analysis of the total number cells loaded in the Boyden chamber verses total number of cells migrated towards the chemo attractant (10% FBS) in each case are shown in D–F. (G) Western blot for IOSE 7576, IOSE 397 and SKOV3 cell lines confirming knock-down of FST by FST-siRNA.

To determine whether FST may be directly contributing to cell motility, we used an FST knock-down approach using FST-siRNA and monitor its effect on cell migration. As shown in Figure 9A–F, cell migration was significantly (P<0.05) decreased not only in IOSE 7576 and IOSE 397 cells that were treated with FST-siRNA but also in parental SKOV3 cells treated with FST-siRNA when compared to their respective controls. Figure 9A–C shows the direct end-point analysis done by lysing of all the migrated cells in each case followed by DNA staining and fluorometric analysis. In contrast, Figure 9D–F demonstrates the number of cells loaded verses number of cells migrated in each case. As loading for most of the samples were more or less same, the difference in the migratory number of the cells reflects the treated/untreated conditions for each of the cell lines. We further evaluated that the decrease in cellular migration was not due to change in morphology of the cells; phase contrast micrographs from all the cells transfected with control siRNA and FST-siRNA did not reveal any change in morphology (Figure S2). The efficiency of FST knock-down in IOSE and SKOV3 cell lines, as detected by Western blotting, is shown in Figure 9G.

## Discussion

FST, initially identified in ovarian follicular fluid, was shown to play a role in cell growth, differentiation and development of ovarian cells [39], [40]. FST is also shown to regulate tumor progression, angiogenesis, metastasis and apoptosis [11], [41], [42], and acts as an antagonist to activin function. Activin is frequently overexpressed in many cancers including ovarian carcinogenesis [43], [44]. Families with history of breast cancer have 50% increased risk of developing ovarian cancer due to germ line mutations in either *BRCA1* or *BRCA2* tumor suppressor genes. *BRCA1*, in particular, plays a major role in hereditary predisposition for breast and ovarian cancer susceptibility [45], [46]. Even though, the role of BRCA1 has been established in cases of hereditary breast and ovarian cancer, its function in ovarian surface epithelial (OSE) cells needs further assessment. The relation between the OSE cells and its link to ovarian cancer has expanded recently due to the ability of cultured OSE to give rise to an ovarian adenocarcinoma in experimental models [47].

Although FST is mainly found in follicular fluids, its expression has been detected in many tissues including ovary, kidney, brain, testis, pancreas, gut, heart, uterus, skeletal muscle, lung, breast, prostate and pituitary indicating that the function of FST may not only be confined to the reproductive system [48], [49]. BRCA1 is also expressed at variable levels in a wide range of normal and pathological human tissues, but mostly it was investigated in relation to tumor suppression in breast, prostate and ovarian cancer. Hypothetically, FST overexpression could be beneficial in treating ovarian tumors with high expression of Activin. Ectopic expression of BRCA1 in ovarian cells may induce the expression of FST and this FST expression in turn plays an antagonistic role to inhibit the cancer promoting function of activin. This phenomenon may very well exist in breast and prostate too, but it needs further investigation. Although this hypothesis may not work with tumor tissues where FST expression is high when compared to its normal expression such as in rodent liver tumors [50], but BRCA1 may offer a tumor suppressive function in the tumors with high activin expression such as human ovarian cancer.

Several key proteins such as activin, inhibin and FST, besides the hormones secreted by the pituitary gland, regulate the process of folliculogenesis. Moreover, activin treatment in ovarian cancer cell lines was shown to induce increased cell proliferation, whereas simultaneous FST treatment in such activin producing cell lines inhibits cell multiplication [10], [51]. Activin is shown to play a complex role in cancer progression and requires careful interpretation with respect to tumorigenesis. Activin effects are cell-type specific such as proliferative effect of activin is observed in cultured tumor epithelial cells obtained from ovarian carcinoma specimens [10], whereas in breast cancer cell line, MCF-7, activin slows the growth of breast tumor cells by inducing G0/G1 cell cycle arrest [52]. We studied mRNA expression of BRCA1, FST, Activin, and SMAD6 proteins in SKOV3 and IOSE cells as well as investigated the ability of these cells to secrete FST in culture medium with respect to BRCA1 expression. It is important to note that IOSE cells were of significant interest since majority of ovarian cancers arise in ovarian surface epithelium. IOSE 592F cells isolated from an ovarian cancer patient with a deletion mutation in *BRCA1* secreted significantly low levels of FST in the medium compared to IOSE 7576 and IOSE 397. The TGF-β superfamily members, activin and its antagonist FST, act as a pleiotropic growth factor system that controls cell proliferation, differentiation, and apoptosis of numerous cell types [10], [51], [53], [54]. Activin is regulated by TGF-ß members and is a known stimulator of ovarian carcinogenesis [55], [56]. These results indicate a novel role for the tumor suppressor BRCA1 in ovarian carcinogenesis.

SMAD6, an inhibitory SMAD member of TGF-ß family of proteins, exert an inhibitory effect towards the stimulatory action of the TGF-ß signaling pathway proteins [57]. Activin, TGF-β1 and BMP-7 are some of the TGF-ß family members known to modulate SMAD6 expression [58]. Polymorphism study utilizing ovarian tissues from ovarian cancer patients reveal that mutations in SMAD6 gene are unlikely to be involved in ovarian carcinogenesis [59]. However, studies with human SMAD6 demonstrate that SMAD6 partially inhibits the function of activin during mesoderm formation [60]. Moreover, ectopic expression of SMAD6 in xenopus embryos was able to entirely block the effects of BMP-4 signaling [61] leading to developmental defects. It is known that activin receptor like kinase 2 (ALK2) interacts with Activin as well as BMPII and TBRII receptors [62], and these ALK2 interactions are sufficient to induce epithelial-mesenchymal transmission (EMT) in heart. SMAD6, which is downstream of ALK2, inhibits EMT in endocardial cells [63] underlining the importance of SMAD6 in cancer development [26]. Additional work is required to link the function of SMAD6 with FST in relation to human ovarian carcinogenesis. Our microarray results suggest that BRCA1 selectively modulates SMAD6 expression in ovarian cancer cells. Although there are no reports showing involvement of SMAD6 in ovarian cancer, the stimulation of SMAD6 by BRCA1 as observed in our study may be suggestive of cytoprotective role of SMAD6 in ovarian cancer, and thus needs further investigation.

To date, regulation of *FST* gene is not well established. Recently, bone morphogenetic protein 2 (BMP-2) and forkhead domain transcription factor L2 (FOXL2) were shown to regulate FST expression in mouse ovary. Moreover, FST expression was also shown to be reduced in Wnt4 null mouse during early ovarian development suggesting positive regulation of *FST* gene by BMP2, FOXL2 and Wnt4 [64]. In addition, expression of FST has been shown to inhibit the up-regulation of Sp6, which is a member of the Sp family of transcription factors that regulate a wide range of cellular functions in ameloblasts [65]. FOXL2 is known as a candidate modulator of smad binding element (SBE1) function. FOXL2 binds to SMAD3 but not to SMAD2 or SMAD4 [66] and its association with SMAD6 is not yet established. FST might modulate ovarian functions by interacting with other family members of TGF-ß superfamily, especially with bone morphogenetic proteins (BMPs). FST interacts with GDF-9, BMP-15, BMP-6, BMP-4, and BMP-7 within ovarian cells [67], [68]. BMPs are involved in contradictory roles of cancer inhibition as well as cancer progression. BRCA1 may induce its cytoprotective effect via modulating the expression of both SMAD6 and FST [69], and in turn, may induce BMP signaling pathways, which may then inhibit or delay the progression of ovarian cancer, specifically epithelial-origin ovarian cancer.

Cellular proliferation, migration, and differentiation are critical functions of cells that in part, assist in tissue rebuilding and repair. Activin has been shown to inhibit cell proliferation but promote cell migration [70], [71]. As an antagonist of activin function, FST expression in cells will have an immense potential in regulating normal cellular homeostasis. Although FST originally has been found in the ovarian follicular fluid, recent reports implicate its role in the regulation of various types of cancer development and/or prevention. In addition, FST was also shown to be expressed in the migrating endothelial cells and simultaneously induce basic fibroblast growth factor (bFGF), thus demonstrating its role in functional regulation of endothelial cells [72]. Interplay between wound healing, angiogenesis and remodeling of molecular matrix serves as an important step for the progression of any cancer including ovarian carcinogenesis. Our data demonstrate that down-regulation of FST leads to reduced cell migration (less wound healing) when compared with the experimental controls. It may interfere with the normal cellular process and normal wound healing, delayed or incomplete, and thus leading to other factors that eventually determine the oncogenic transformation of the cells. Studies using prostate and liver tumors show that genes involving wound healing were highly activated, whereas tumors originated from breast and colon cancer displayed mixed results with regards to the activation of wound healing genes [73]. Role of angiogenesis has been well documented in the progression of tumor growth and metastasis. Reports suggest that FST overexpression leads to induction of angiogenesis and simultaneously demonstrate the inhibitory action on Activin function in ovarian carcinogenesis. FST overexpressing tumors showed reduced tumor growth when compared with mock control [42]. Thus, additional *in-vivo* experiments will allow us to justify the possible role of FST as regulator of ovarian carcinogenesis. In this manuscript, we have shown that BRCA1 induces FST expression in SKOV3 and IOSE cells and this FST induction may be mediated by several other potential genes such as *FOXL2*, *BMP-2*, and *Sp6*. Moreover, BRCA1 induced FST modulation may be tissue-specific such as human ovarian tissue. Finally, additional studies specifically examining the interplay between BRCA1 and FST and its effect(s) on cellular processes may be helpful in devising targeted treatment for human ovarian cancer.

## Supporting Information

Figure S1 **Hierarchical gene clustering generated by the Ingenuity Pathway Knowledge Base.** A heat map showing significant (P<0.05) changes in a group of 218 genes where the genes were either up-regulated more than 10 fold or genes were down-regulated more than 4 fold due to stable overexpression of BRCA1 in SKOV3 cells (compared to empty pcDNA3 stable lines). Three independent microarray analyses were performed.
(TIF)

Figure S2 **Effect of FST-siRNA treatment on cell morphology.** IOSE and SKOV3 cells were transfected with either control siRNA or FST-siRNA and phase contrast images were captured through a light microscope using 63X objective. All photomicrographs are labeled with the cells used in this study. No change in cell morphology is observed between control-siRNA and FST-siRNA treated cells for all cell types.
(TIF)

Table S1 **Up-regulation of Genes Due to BRCA1 overexpression.**
(DOC)

Table S2 **Pathway specific up-regulation of the genes with the up-regulation of BRCA1 in SKOV3 cells.**
(DOC)

Table S3 **Pathway specific down-regulation of genes with the up-regulation of BRCA1 in SKOV3 cells.**
(DOC)
