# Supplementary figures and images for: BRCA1 Regulates Follistatin Function in Ovarian Cancer and Human Ovarian Surface Epithelial Cells
Source: PLoS One. 2012 Jun 1;7(6):e37697. doi: 10.1371/journal.pone.0037697 (PMC3365892; doi:10.1371/journal.pone.0037697)

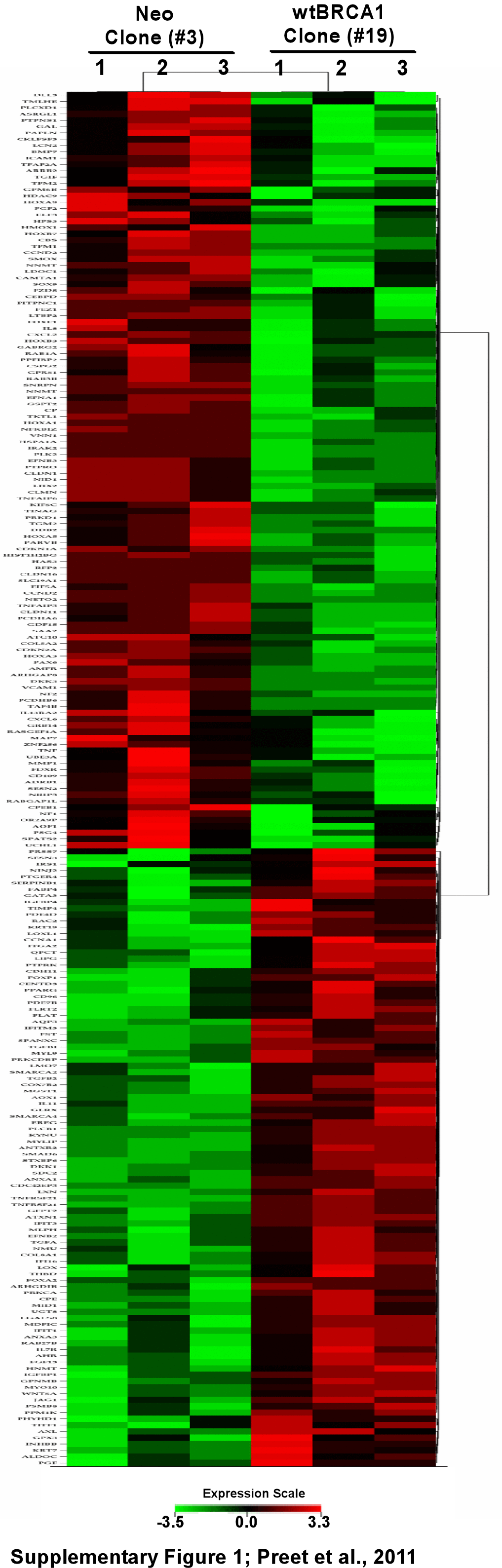

Supplement: Figure S1 — Hierarchical gene clustering generated by the Ingenuity Pathway Knowledge Base. A heat map showing significant (P<0.05) changes in a group of 218 genes where the genes were either up-regulated more than 10 fold or genes were down-regulated more than 4 fold due to stable overexpression of BRCA1 in SKOV3 cells (compared to empty pcDNA3 stable lines). Three independent microarray analyses were performed. (TIF) [file pone.0037697.s001.tif]

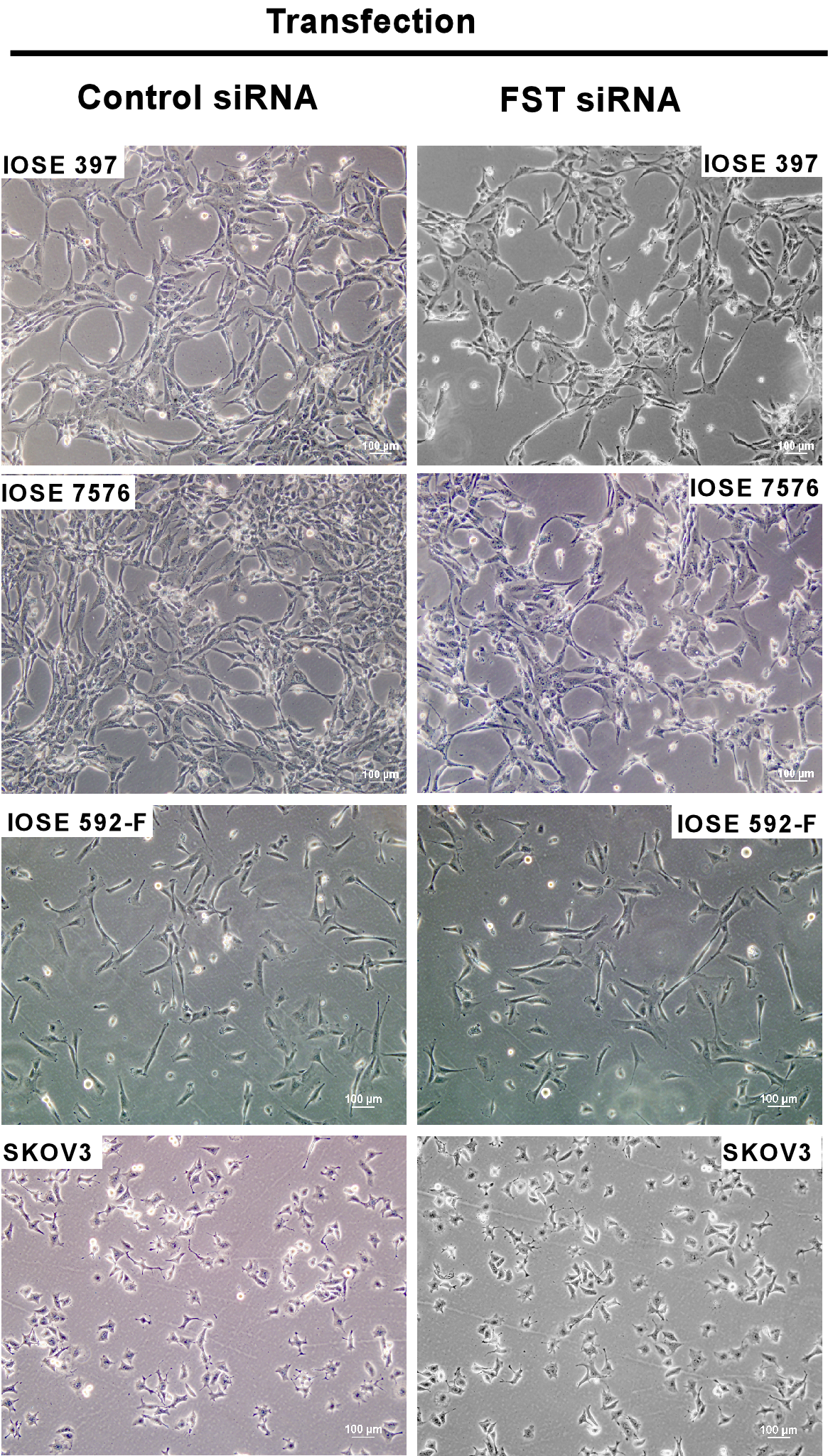

Supplement: Figure S2 — Effect of FST-siRNA treatment on cell morphology. IOSE and SKOV3 cells were transfected with either control siRNA or FST-siRNA and phase contrast images were captured through a light microscope using 63X objective. All photomicrographs are labeled with the cells used in this study. No change in cell morphology is observed between control-siRNA and FST-siRNA treated cells for all cell types. (TIF) [file pone.0037697.s002.tif]
