# Supplementary material for: BRCA1 Regulates Follistatin Function in Ovarian Cancer and Human Ovarian Surface Epithelial Cells
Source: PLoS One. 2012 Jun 1;7(6):e37697. doi: 10.1371/journal.pone.0037697 (PMC3365892; doi:10.1371/journal.pone.0037697)
Supplement: Table S1 — Up-regulation of Genes Due to BRCA1 overexpression. (DOC) [file pone.0037697.s003.doc]

**Table S1**:

*Up-regulation of Genes Due to BRCA1 overexpression*

| **Number** | **Symbol** | **GenBank** | **Description** | **Fold** | **p-value** |
| --- | --- | --- | --- | --- | --- |
| 1 | EDIL3 | AA053711 | EGF-like repeats and discoidin I-like domains 3 | 290.1 | 0.000146 |
| 2 | GPR116 | BF941499 | G protein-coupled receptor 116 | 265.1 | 5.28E-05 |
| 3 | DUSP4 | NM_001394 | dual specificity phosphatase 4 | 255.1 | 7.78E-09 |
| 4 | CDH11 | D21254 | cadherin 11, type 2, OB-cadherin (osteoblast) | 167.4 | 3.33E-06 |
| 5 | AMIGO2 | AC004010 | adhesion molecule with Ig-like domain 2 | 151.5 | 1.03E-07 |
| 6 | SUSD2 | Z92546 | sushi domain containing 2 | 131.9 | 0.0006502 |
| 7 | ARHGDIB | NM_001175 | Rho GDP dissociation inhibitor (GDI) beta | 119.2 | 0.0002288 |
| 8 | ARHGDIB | AF498927 | Rho GDP dissociation inhibitor (GDI) beta | 111.5 | 0.0001694 |
| 9 | SPANXA1 | NM_013453 | sperm protein associated with the nucleus, X-linked, family member A1 | 97.3 | 0.0001174 |
| 10 | AXL | NM_021913 | AXL receptor tyrosine kinase | 96.5 | 0.0001398 |
| 11 | VAMP8 | NM_003761 | vesicle-associated membrane protein 8 (endobrevin) | 92.9 | 4.94E-05 |
| 12 | SDPR | BF982174 | serum deprivation response (phosphatidylserine binding protein) | 91.3 | 1.68E-07 |
| 13 | COX7B2 | AI126453 | cytochrome c oxidase subunit VIIb2 | 90.7 | 1.55E-06 |
| 14 | DKK1 | NM_012242 | dickkopf homolog 1 (Xenopus laevis) | 83.4 | 4.81E-07 |
| 15 | AIM1 | U83115 | absent in melanoma 1 | 72.9 | 9.24E-06 |
| 16 | FGF13 | NM_004114 | fibroblast growth factor 13 | 71.8 | 1.41E-06 |
| 17 | AQP3 | N74607 | aquaporin 3 | 71.7 | 4.73E-05 |
| 18 | GCNT2 | BF059748 | glucosaminyl (N-acetyl) transferase 2, I-branching enzyme | 71.2 | 0.0001242 |
| 19 | TGFB2 | M19154 | transforming growth factor, beta 2 | 70.8 | 3.67E-06 |
| 20 | MAGEA2B | U82671 | melanoma antigen family A, 2B | 69.5 | 0.0007062 |
| 21 | PRKCDBP | AI088622 | protein kinase C, delta binding protein | 66.3 | 5.68E-07 |
| 22 | PLAC8 | NM_016619 | placenta-specific 8 | 66.2 | 6.70E-07 |
| 23 | KIAA1199 | AB033025 | KIAA1199 | 65.3 | 1.08E-05 |
| 24 | GJA1 | NM_000165 | gap junction protein, alpha 1, 43kDa (connexin 43) | 64.6 | 2.13E-06 |
| 25 | GPR116 | N95226 | G protein-coupled receptor 116 | 64.3 | 3.61E-05 |
| 26 | WNT5A | NM_003392 | wingless-type MMTV integration site family, member 5A | 60.9 | 1.60E-05 |
| 27 | TMEM71 | AI342543 | transmembrane protein 71 | 60.4 | 3.94E-05 |
| 28 | GBP3 | AL136680 | guanylate binding protein 3 | 59.5 | 0.0002036 |
| 29 | COL13A1 | M33653 | collagen, type XIII, alpha 1 | 57.9 | 1.69E-05 |
| 30 | IFIT3 | AI075407 | interferon-induced protein with tetratricopeptide repeats 3 | 55.4 | 1.49E-06 |
| 31 | IGFBP4 | NM_001552 | insulin-like growth factor binding protein 4 | 54.9 | 6.60E-05 |
| 32 | HNMT | NM_006895 | histamine N-methyltransferase | 54.3 | 0.0004093 |
| 33 | MARCKS | AI709406 | myristoylated alanine-rich protein kinase C substrate | 53.3 | 8.24E-05 |
| 34 | AGR2 | AF088867 | anterior gradient 2 homolog (Xenopus laevis) | 53.0 | 4.19E-06 |
| 35 | MYLIP | AW292746 | myosin regulatory light chain interacting protein | 52.6 | 3.81E-05 |
| 36 | FST | BF438173 | follistatin | 51.6 | 2.34E-05 |
| 37 | CLEC2B | CA447397 | C-type lectin domain family 2, member B | 50.5 | 1.36E-06 |
| 38 | DUSP4 | BC002671 | dual specificity phosphatase 4 | 50.2 | 1.43E-06 |
| 39 | ARMCX1 | NM_016608 | armadillo repeat containing, X-linked 1 | 49.9 | 0.0005025 |
| 40 | SAMD9 | AA741307 | sterile alpha motif domain containing 9 | 49.6 | 2.88E-06 |
| 41 | F2RL1 | BE965369 | coagulation factor II (thrombin) receptor-like 1 | 49.1 | 3.00E-07 |
| 42 | ETV1 | BE881590 | ets variant gene 1 | 49.0 | 2.51E-05 |
| 43 | CLEC2B | BC005254 | C-type lectin domain family 2, member B | 48.8 | 7.69E-05 |
| 44 | KRT19 | NM_002276 | keratin 19 | 47.8 | 0.0006476 |
| 45 | DAB2 | NM_001343 | disabled homolog 2, mitogen-responsive phosphoprotein (Drosophila) | 46.9 | 1.16E-06 |
| 46 | STXBP6 | N21096 | syntaxin binding protein 6 (amisyn) | 46.8 | 3.10E-05 |
| 47 | ANXA3 | M63310 | annexin A3 | 45.8 | 1.67E-05 |
| 48 | FOXP1 | AK026898 | forkhead box P1 | 45.6 | 8.32E-07 |
| 49 | MAGEA2 | NM_005361 | melanoma antigen family A, 2 | 45.5 | 3.78E-07 |
| 50 | SMARCA2 | AV725365 | SWI/SNF related, matrix associated, actin dependent regulator of chromatin, subfamily a, member 2 | 44.4 | 4.66E-06 |
| 51 | F2RL1 | NM_005242 | coagulation factor II (thrombin) receptor-like 1 | 44.0 | 0.000143 |
| 52 | TCEAL8 | AI743979 | transcription elongation factor A (SII)-like 8 | 44.0 | 0.0002187 |
| 53 | MARCKS | M68956 | myristoylated alanine-rich protein kinase C substrate | 43.4 | 1.06E-05 |
| 54 | JAG1 | U77914 | jagged 1 (Alagille syndrome) | 42.1 | 0.0001236 |
| 55 | MARCKS | NM_002356 | myristoylated alanine-rich protein kinase C substrate | 41.5 | 5.16E-07 |
| 56 | RAC2 | BE138888 | ras-related C3 botulinum toxin substrate 2 (rho family, small GTP binding protein Rac2) | 41.0 | 9.88E-05 |
| 57 | FAM46C | AL046017 | family with sequence similarity 46, member C | 40.9 | 3.06E-05 |
| 58 | EDG2 | BF055366 | endothelial differentiation, lysophosphatidic acid G-protein-coupled receptor, 2 | 40.6 | 0.0002864 |
| 59 | SMARCA2 | AI535683 | SWI/SNF related, matrix associated, actin dependent regulator of chromatin, subfamily a, member 2 | 40.0 | 0.0001885 |
| 60 | SLCO1B3 | NM_019844 | solute carrier organic anion transporter family, member 1B3 | 39.6 | 2.70E-05 |
| 61 | KYNU | D55639 | kynureninase (L-kynurenine hydrolase) | 39.5 | 1.93E-06 |
| 62 | QPCT | NM_012413 | glutaminyl-peptide cyclotransferase (glutaminyl cyclase) | 38.6 | 8.60E-05 |
| 63 | FOXP1 | AF146696 | forkhead box P1 | 38.3 | 9.84E-05 |
| 64 | FOXA2 | AB028021 | forkhead box A2 | 36.5 | 0.0002231 |
| 65 | C1orf139 | AL534095 | chromosome 1 open reading frame 139 | 36.2 | 1.12E-05 |
| 66 | IL11 | NM_000641 | interleukin 11 | 36.2 | 9.30E-06 |
| 67 | TGFB2 | AU145950 | transforming growth factor, beta 2 | 35.5 | 5.44E-05 |
| 68 | NEDD9 | AL136139 | neural precursor cell expressed, developmentally down-regulated 9 | 35.1 | 7.47E-06 |
| 69 | SPARC | NM_003118 | secreted protein, acidic, cysteine-rich (osteonectin) | 34.7 | 0.0001618 |
| 70 | WNT5A | AI968085 | wingless-type MMTV integration site family, member 5A | 34.3 | 6.72E-05 |
| 71 | IFIT1 | NM_001548 | interferon-induced protein with tetratricopeptide repeats 1 | 33.9 | 7.07E-06 |
| 72 | F3 | NM_001993 | coagulation factor III (thromboplastin, tissue factor) | 33.6 | 0.0001981 |
| 73 | MAGEB2 | NM_002364 | melanoma antigen family B, 2 | 33.6 | 2.61E-05 |
| 74 | FABP4 | NM_001442 | fatty acid binding protein 4, adipocyte | 32.8 | 0.0005695 |
| 75 | PMP22 | L03203 | peripheral myelin protein 22 | 32.0 | 0.0001356 |
| 76 | PTPRK | NM_002844 | protein tyrosine phosphatase, receptor type, K | 31.9 | 2.79E-05 |
| 77 | SERPINB1 | AI554300 | serpin peptidase inhibitor, clade B (ovalbumin), member 1 | 31.8 | 0.0001676 |
| 78 | SLC43A3 | AI630178 | solute carrier family 43, member 3 | 31.8 | 0.0004543 |
| 79 | SDPR | NM_004657 | serum deprivation response (phosphatidylserine binding protein) | 31.6 | 0.0004477 |
| 80 | GALNT3 | BF063271 | UDP-N-acetyl-alpha-D-galactosamine:polypeptide N-acetylgalactosaminyltransferase 3 (GalNAc-T3) | 30.5 | 2.33E-05 |
| 81 | C1orf139 | AA775681 | chromosome 1 open reading frame 139 | 30.5 | 1.98E-06 |
| 82 | IFITM3 | BF338947 | interferon induced transmembrane protein 3 (1-8U) | 30.2 | 1.28E-06 |
| 83 | BDNF | BF674612 | brain-derived neurotrophic factor | 30.0 | 1.47E-05 |
| 84 | IRS1 | NM_005544 | insulin receptor substrate 1 | 30.0 | 0.0004874 |
| 85 | SNAI2 | AI572079 | snail homolog 2 (Drosophila) | 29.7 | 0.0006777 |
| 86 | NAV2 | NM_018162 | neuron navigator 2 | 29.5 | 7.48E-05 |
| 87 | PXDN | D86983 | peroxidasin homolog (Drosophila) | 29.3 | 6.48E-05 |
| 88 | RCSD1 | AI659418 | RCSD domain containing 1 | 28.4 | 2.42E-06 |
| 89 | RBM24 | AI677701 | RNA binding motif protein 24 | 28.3 | 0.0001851 |
| 90 | NOV | BF440025 | nephroblastoma overexpressed gene | 27.9 | 0.0004883 |
| 91 | CPE | NM_001873 | carboxypeptidase E | 27.7 | 1.64E-06 |
| 92 | TNFRSF21 | BE568134 | tumor necrosis factor receptor superfamily, member 21 | 27.6 | 3.97E-07 |
| 93 | MYL9 | NM_006097 | myosin, light polypeptide 9, regulatory | 27.4 | 1.43E-06 |
| 94 | AKR1C3 | AB018580 | aldo-keto reductase family 1, member C3 (3-alpha hydroxysteroid dehydrogenase, type II) | 27.4 | 0.0001823 |
| 95 | ANXA1 | NM_000700 | annexin A1 | 27.1 | 7.70E-08 |
| 96 | HBE1 | NM_005330 | hemoglobin, epsilon 1 | 26.6 | 0.0002207 |
| 97 | C1orf139 | AL534095 | chromosome 1 open reading frame 139 | 26.5 | 1.55E-06 |
| 98 | SPARC | AL575922 | secreted protein, acidic, cysteine-rich (osteonectin) | 26.4 | 3.05E-06 |
| 99 | FOXA2 | AB028021 | forkhead box A2 | 26.4 | 2.24E-06 |
| 100 | KRT7 | BC002700 | keratin 7 | 26.3 | 0.0006267 |
| 101 | IFIT2 | AA131041 | interferon-induced protein with tetratricopeptide repeats 2 | 25.9 | 7.18E-06 |
| 102 | KCTD12 | AI718937 | potassium channel tetramerisation domain containing 12 | 25.8 | 5.62E-05 |
| 103 | LXN | NM_020169 | latexin | 25.8 | 2.39E-06 |
| 104 | ACSL5 | NM_016234 | acyl-CoA synthetase long-chain family member 5 | 25.6 | 6.18E-06 |
| 105 | IFITM2 | NM_006435 | interferon induced transmembrane protein 2 (1-8D) | 25.6 | 8.97E-07 |
| 106 | IFI16 | NM_005531 | interferon, gamma-inducible protein 16 | 25.5 | 4.45E-05 |
| 107 | GPR110 | BG426455 | G protein-coupled receptor 110 | 25.3 | 8.36E-05 |
| 108 | SMARCA4 | D26156 | SWI/SNF related, matrix associated, actin dependent regulator of chromatin, subfamily a, member 4 | 25.3 | 7.66E-05 |
| 109 | RAC2 | NM_002872 | ras-related C3 botulinum toxin substrate 2 (rho family, small GTP binding protein Rac2) | 25.2 | 4.90E-06 |
| 110 | ATXN1 | NM_000332 | ataxin 1 | 25.0 | 3.93E-06 |
| 111 | JAG1 | U73936 | jagged 1 (Alagille syndrome) | 24.9 | 9.48E-07 |
| 112 | GBP1 | AW014593 | guanylate binding protein 1, interferon-inducible, 67kDa | 24.7 | 6.51E-06 |
| 113 | KITLG | AI446414 | KIT ligand | 24.7 | 2.22E-05 |
| 114 | MID1 | NM_000381 | midline 1 (Opitz/BBB syndrome) | 24.5 | 2.82E-07 |
| 115 | TXNIP | NM_006472 | thioredoxin interacting protein | 24.5 | 0.0007499 |
| 116 | ANTXR2 | BE673665 | anthrax toxin receptor 2 | 24.5 | 1.76E-05 |
| 117 | TNFRSF21 | NM_016629 | tumor necrosis factor receptor superfamily, member 21 | 24.5 | 8.76E-07 |
| 118 | SMARCA2 | NM_003070 | SWI/SNF related, matrix associated, actin dependent regulator of chromatin, subfamily a, member 2 | 24.3 | 3.37E-05 |
| 119 | PDE4B | NM_002600 | phosphodiesterase 4B, cAMP-specific (phosphodiesterase E4 dunce homolog, Drosophila) | 24.2 | 7.81E-05 |
| 120 | PDE7B | AI638433 | phosphodiesterase 7B | 24.0 | 1.27E-05 |
| 121 | COL8A1 | AL359062 | collagen, type VIII, alpha 1 | 24.0 | 2.34E-05 |
| 122 | TITF1 | BC006221 | thyroid transcription factor 1 | 23.6 | 4.24E-05 |
| 123 | MGST1 | D16947 | microsomal glutathione S-transferase 1 | 23.1 | 3.47E-07 |
| 124 | DAF | BC001288 | decay accelerating factor for complement (CD55, Cromer blood group system) | 23.1 | 1.18E-06 |
| 125 | S100A4 | NM_002961 | S100 calcium binding protein A4 (calcium protein, calvasculin, metastasin, murine placental homolog) | 23.1 | 0.0001986 |
| 126 | PTGER4 | AA897516 | prostaglandin E receptor 4 (subtype EP4) | 23.1 | 6.14E-05 |
| 127 | EVI2A | NM_014210 | ecotropic viral integration site 2A | 22.9 | 0.000142 |
| 128 | IFI16 | AF208043 | interferon, gamma-inducible protein 16 | 22.9 | 3.05E-05 |
| 129 | DAF | CA448665 | decay accelerating factor for complement (CD55, Cromer blood group system) | 22.7 | 7.98E-05 |
| 130 | LAPTM5 | NM_006762 | lysosomal associated multispanning membrane protein 5 | 22.6 | 2.10E-06 |
| 131 | IFI44 | NM_006417 | interferon-induced protein 44 | 22.2 | 1.54E-05 |
| 132 | PFTK1 | NM_012395 | PFTAIRE protein kinase 1 | 22.2 | 0.0002948 |
| 133 | TGFB2 | NM_003238 | transforming growth factor, beta 2 | 22.0 | 1.19E-05 |
| 134 | HNMT | BC005907 | histamine N-methyltransferase | 21.8 | 1.11E-05 |
| 135 | DAF | NM_000574 | decay accelerating factor for complement (CD55, Cromer blood group system) | 21.8 | 1.63E-06 |
| 136 | PLSCR4 | NM_020353 | phospholipid scramblase 4 | 21.6 | 9.34E-05 |
| 137 | C1orf85 | BF977145 | chromosome 1 open reading frame 85 | 21.4 | 3.30E-06 |
| 138 | ARID5B | BG285011 | AT rich interactive domain 5B (MRF1-like) | 21.4 | 1.19E-05 |
| 139 | SDC2 | AL577322 | syndecan 2 (heparan sulfate proteoglycan 1, cell surface-associated, fibroglycan) | 21.1 | 2.04E-05 |
| 140 | CDH11 | AI754693 | cadherin 11, type 2, OB-cadherin (osteoblast) | 20.9 | 9.87E-06 |
| 141 | FLRT2 | NM_013231 | fibronectin leucine rich transmembrane protein 2 | 20.8 | 1.36E-05 |
| 142 | DAB2 | N21202 | disabled homolog 2, mitogen-responsive phosphoprotein (Drosophila) | 20.8 | 1.94E-06 |
| 143 | PLAT | NM_000930 | plasminogen activator, tissue | 20.7 | 1.71E-05 |
| 144 | NTN4 | AF278532 | netrin 4 | 20.2 | 0.0001112 |
| 145 | PGF | BC001422 | placental growth factor, vascular endothelial growth factor-related protein | 20.1 | 6.87E-05 |
| 146 | SLC43A3 | BC003163 | solute carrier family 43, member 3 | 19.9 | 0.0006195 |
| 147 | LGALS8 | L78132 | lectin, galactoside-binding, soluble, 8 (galectin 8) | 19.7 | 0.0003405 |
| 148 | IGFBP1 | NM_000596 | insulin-like growth factor binding protein 1 | 19.7 | 7.89E-05 |
| 149 | ABCA8 | NM_007168 | ATP-binding cassette, sub-family A (ABC1), member 8 | 19.6 | 3.00E-05 |
| 150 | BCL6 | NM_001706 | B-cell CLL/lymphoma 6 (zinc finger protein 51) | 19.2 | 0.0001195 |
| 151 | SDC2 | AI380298 | syndecan 2 (heparan sulfate proteoglycan 1, cell surface-associated, fibroglycan) | 19.2 | 0.0001049 |
| 152 | HEATR1 | AI659005 | HEAT repeat containing 1 | 19.0 | 3.50E-07 |
| 153 | TRIB2 | NM_021643 | tribbles homolog 2 (Drosophila) | 18.9 | 0.0004838 |
| 154 | PPARG | NM_015869 | peroxisome proliferative activated receptor, gamma | 18.9 | 4.28E-05 |
| 155 | PLCB1 | AL049593 | phospholipase C, beta 1 (phosphoinositide-specific) | 18.8 | 6.01E-05 |
| 156 | AHR | NM_001621 | aryl hydrocarbon receptor | 18.7 | 5.01E-06 |
| 157 | GPX3 | NM_002084 | glutathione peroxidase 3 (plasma) | 18.7 | 5.75E-05 |
| 158 | LGALS3BP | NM_005567 | lectin, galactoside-binding, soluble, 3 binding protein | 18.6 | 1.10E-05 |
| 159 | DAB2 | AF188298 | disabled homolog 2, mitogen-responsive phosphoprotein (Drosophila) | 18.6 | 5.17E-05 |
| 160 | SLC44A5 | AA001450 | solute carrier family 44, member 5 | 18.5 | 0.000156 |
| 161 | MID1 | BE967532 | midline 1 (Opitz/BBB syndrome) | 18.0 | 4.44E-05 |
| 162 | GPC6 | AI651255 | glypican 6 | 18.0 | 0.0001392 |
| 163 | TGFA | NM_003236 | transforming growth factor, alpha | 17.5 | 5.92E-06 |
| 164 | GPNMB | NM_002510 | glycoprotein (transmembrane) nmb | 17.5 | 2.11E-06 |
| 165 | CD274 | AI608902 | CD274 antigen | 17.4 | 0.0002549 |
| 166 | LOXL1 | NM_005576 | lysyl oxidase-like 1 | 17.3 | 3.53E-05 |
| 167 | SMARCA4 | AI831675 | SWI/SNF related, matrix associated, actin dependent regulator of chromatin, subfamily a, member 4 | 17.3 | 4.98E-07 |
| 168 | SERPINB1 | NM_030666 | serpin peptidase inhibitor, clade B (ovalbumin), member 1 | 17.0 | 0.0006289 |
| 169 | CD96 | NM_005816 | CD96 antigen | 17.0 | 4.40E-06 |
| 170 | GLRX | AF162769 | glutaredoxin (thioltransferase) | 17.0 | 0.0002097 |
| 171 | SESN3 | BE883841 | sestrin 3 | 17.0 | 0.0003643 |
| 172 | LMO7 | AA100793 | LIM domain 7 | 16.9 | 6.09E-05 |
| 173 | GMFG | NM_004877 | glia maturation factor, gamma | 16.6 | 0.0001612 |
| 174 | SLAMF7 | AL121985 | SLAM family member 7 | 16.5 | 0.000254 |
| 175 | TRIM38 | AU157590 | tripartite motif-containing 38 | 16.4 | 1.24E-05 |
| 176 | PAG1 | AK000680 | phosphoprotein associated with glycosphingolipid microdomains 1 | 16.4 | 1.75E-05 |
| 177 | NT5E | NM_002526 | 5'-nucleotidase, ecto (CD73) | 16.4 | 1.50E-06 |
| 178 | ANTXR2 | AU152178 | anthrax toxin receptor 2 | 16.4 | 5.02E-05 |
| 179 | CCNA1 | NM_003914 | cyclin A1 | 16.3 | 0.0002256 |
| 180 | MGST1 | NM_020300 | microsomal glutathione S-transferase 1 | 16.3 | 2.00E-05 |
| 181 | GBP2 | BF509371 | guanylate binding protein 2, interferon-inducible | 16.2 | 0.0002757 |
| 182 | PSMB8 | U17496 | proteasome (prosome, macropain) subunit, beta type, 8 (large multifunctional peptidase 7) | 16.2 | 1.06E-05 |
| 183 | PPM1K | AV706522 | protein phosphatase 1K (PP2C domain containing) | 16.2 | 4.93E-05 |
| 184 | MYO10 | NM_012334 | myosin X | 16.1 | 3.55E-06 |
| 185 | SMARCA4 | AK026573 | SWI/SNF related, matrix associated, actin dependent regulator of chromatin, subfamily a, member 4 | 16.1 | 0.0001205 |
| 186 | GUCY1B3 | W93728 | guanylate cyclase 1, soluble, beta 3 | 16.1 | 0.0001253 |
| 187 | PRKCA | AI471375 | protein kinase C, alpha | 16.0 | 2.03E-07 |
| 188 | NMU | NM_006681 | neuromedin U | 15.9 | 3.46E-06 |
| 189 | PAPSS2 | AF074331 | 3'-phosphoadenosine 5'-phosphosulfate synthase 2 | 15.9 | 4.04E-07 |
| 190 | GPR158 | R41459 | G protein-coupled receptor 158 | 15.8 | 7.53E-05 |
| 191 | PAPSS2 | AW299958 | 3'-phosphoadenosine 5'-phosphosulfate synthase 2 | 15.8 | 0.000113 |
| 192 | FAM13A1 | NM_014883 | family with sequence similarity 13, member A1 | 15.8 | 3.95E-05 |
| 193 | TIMP4 | NM_003256 | TIMP metallopeptidase inhibitor 4 | 15.7 | 0.0002626 |
| 194 | GFPT2 | NM_005110 | glutamine-fructose-6-phosphate transaminase 2 | 15.7 | 1.04E-06 |
| 195 | ACSL5 | AW173691 | acyl-CoA synthetase long-chain family member 5 | 15.6 | 2.20E-06 |
| 196 | MGST1 | AI220117 | microsomal glutathione S-transferase 1 | 15.5 | 1.24E-07 |
| 197 | CAPG | NM_001747 | capping protein (actin filament), gelsolin-like | 15.4 | 9.72E-06 |
| 198 | GSTM3 | AL527430 | glutathione S-transferase M3 (brain) | 15.2 | 0.0002452 |
| 199 | SLC39A8 | AB040120 | solute carrier family 39 (zinc transporter), member 8 | 14.8 | 5.29E-05 |
| 200 | SATB1 | NM_002971 | special AT-rich sequence binding protein 1 (binds to nuclear matrix/scaffold-associating DNA's) | 14.8 | 1.24E-06 |
| 201 | MDFIC | AF054589 | MyoD family inhibitor domain containing | 14.7 | 0.0001987 |
| 202 | SLC7A11 | AA488687 | solute carrier family 7, (cationic amino acid transporter, y+ system) member 11 | 14.5 | 5.55E-06 |
| 203 | EDIL3 | NM_005711 | EGF-like repeats and discoidin I-like domains 3 | 14.2 | 0.0002375 |
| 204 | CDC42EP3 | AI801777 | CDC42 effector protein (Rho GTPase binding) 3 | 14.1 | 4.35E-06 |
| 205 | MYLIP | AF212221 | myosin regulatory light chain interacting protein | 14.1 | 3.61E-05 |
| 206 | MLPH | NM_024101 | melanophilin | 14.1 | 4.10E-06 |
| 207 | MYLIP | AW292746 | myosin regulatory light chain interacting protein | 14.0 | 0.0001106 |
| 208 | C10orf45 | BC004872 | chromosome 10 open reading frame 45 | 14.0 | 7.98E-05 |
| 209 | UGT8 | N22272 | UDP glycosyltransferase 8 (UDP-galactose ceramide galactosyltransferase) | 13.9 | 3.48E-05 |
| 210 | IL7R | BE217880 | interleukin 7 receptor | 13.8 | 9.32E-06 |
| 211 | TGFBI | NM_000358 | transforming growth factor, beta-induced, 68kDa | 13.7 | 5.25E-05 |
| 212 | C4BPB | NM_000716 | complement component 4 binding protein, beta | 13.5 | 0.0003852 |
| 213 | GPR37 | U87460 | G protein-coupled receptor 37 (endothelin receptor type B-like) | 13.4 | 9.42E-07 |
| 214 | GLRX | NM_002064 | glutaredoxin (thioltransferase) | 13.3 | 1.02E-05 |
| 215 | CPE | AI922855 | carboxypeptidase E | 13.3 | 7.37E-05 |
| 216 | ROR1 | AA284248 | receptor tyrosine kinase-like orphan receptor 1 | 13.3 | 0.0002654 |
| 217 | EREG | NM_001432 | epiregulin | 13.3 | 7.41E-05 |
| 218 | FREM2 | N66307 | FRAS1 related extracellular matrix protein 2 | 13.3 | 2.21E-05 |
| 219 | CLCN4 | AF052117 | chloride channel 4 | 13.1 | 6.45E-05 |
| 220 | CDC42EP3 | AI754416 | CDC42 effector protein (Rho GTPase binding) 3 | 13.0 | 0.0001472 |
| 221 | C10orf45 | AL136885 | chromosome 10 open reading frame 45 | 13.0 | 0.0001603 |
| 222 | IFI16 | BG256677 | interferon, gamma-inducible protein 16 | 12.9 | 0.0007159 |
| 223 | BDNF | NM_001709 | brain-derived neurotrophic factor | 12.8 | 0.0001186 |
| 224 | GPR110 | AA746038 | G protein-coupled receptor 110 | 12.8 | 8.31E-05 |
| 225 | SLC2A3 | NM_006931 | solute carrier family 2 (facilitated glucose transporter), member 3 | 12.4 | 9.84E-05 |
| 226 | RAB27B | BF438386 | RAB27B, member RAS oncogene family | 12.4 | 9.08E-06 |
| 227 | LOX | L16895 | lysyl oxidase | 12.4 | 2.54E-05 |
| 228 | NR3C1 | AI934556 | nuclear receptor subfamily 3, group C, member 1 (glucocorticoid receptor) | 12.2 | 0.0001022 |
| 229 | AOX1 | NM_001159 | aldehyde oxidase 1 | 12.2 | 5.74E-07 |
| 230 | GATA3 | BC003070 | GATA binding protein 3 | 12.1 | 0.0007354 |
| 231 | ITGA2 | N95414 | integrin, alpha 2 (CD49B, alpha 2 subunit of VLA-2 receptor) | 12.1 | 0.0001174 |
| 232 | EVI1 | BG261252 | ecotropic viral integration site 1 | 12.1 | 1.01E-05 |
| 233 | PDGFD | NM_025208 | platelet derived growth factor D | 12.1 | 0.0001849 |
| 234 | KIAA1217 | AL157473 | KIAA1217 | 12.1 | 4.84E-05 |
| 235 | LY96 | NM_015364 | lymphocyte antigen 96 | 12.0 | 3.71E-06 |
| 236 | EDG2 | AI679812 | endothelial differentiation, lysophosphatidic acid G-protein-coupled receptor, 2 | 12.0 | 1.83E-05 |
| 237 | OAS1 | NM_016816 | 2',5'-oligoadenylate synthetase 1, 40/46kDa | 12.0 | 0.0003908 |
| 238 | C1orf85 | AW090182 | chromosome 1 open reading frame 85 | 12.0 | 2.47E-05 |
| 239 | CENTD3 | NM_022481 | centaurin, delta 3 | 11.9 | 9.70E-06 |
| 240 | PDLIM4 | BC003096 | PDZ and LIM domain 4 | 11.8 | 0.0005021 |
| 241 | INHBB | NM_002193 | inhibin, beta B (activin AB beta polypeptide) | 11.6 | 1.36E-05 |
| 242 | SPANXC | NM_022661 | SPANX family, member C | 11.6 | 6.22E-06 |
| 243 | ALDOC | NM_005165 | aldolase C, fructose-bisphosphate | 11.6 | 3.61E-05 |
| 244 | SAMD9 | NM_017654 | sterile alpha motif domain containing 9 | 11.5 | 7.55E-05 |
| 245 | TRIM38 | AI363270 | tripartite motif-containing 38 | 11.4 | 0.0007075 |
| 246 | CFH | X04697 | complement factor H | 11.4 | 3.37E-05 |
| 247 | NINJ2 | NM_016533 | ninjurin 2 | 11.4 | 0.0001353 |
| 248 | ATP1B1 | AI094580 | ATPase, Na+/K+ transporting, beta 1 polypeptide | 11.3 | 1.31E-06 |
| 249 | CLIC3 | NM_004669 | chloride intracellular channel 3 | 11.3 | 0.0001102 |
| 250 | LIPG | NM_006033 | lipase, endothelial | 11.3 | 8.00E-06 |
| 251 | NR2F1 | AI951185 | nuclear receptor subfamily 2, group F, member 1 | 11.2 | 1.65E-05 |
| 252 | TSC22D1 | AK027071 | TSC22 domain family, member 1 | 11.2 | 3.76E-07 |
| 253 | PHYHD1 | AL545998 | phytanoyl-CoA dioxygenase domain containing 1 | 10.9 | 0.0005268 |
| 254 | FKBP7 | AA683602 | FK506 binding protein 7 | 10.9 | 3.59E-05 |
| 255 | HNMT | AU157303 | histamine N-methyltransferase | 10.9 | 0.0005259 |
| 256 | THBD | NM_000361 | thrombomodulin | 10.8 | 0.000436 |
| 258 | EFNB2 | BF001670 | ephrin-B2 | 10.7 | 4.28E-06 |
| 259 | SMAD6 | NM_005585 | SMAD, mothers against DPP homolog 6 (Drosophila) | 10.7 | 6.41E-07 |
| 260 | MCAM | M28882 | melanoma cell adhesion molecule | 10.6 | 2.41E-06 |
| 261 | TRIB2 | BC002637 | tribbles homolog 2 (Drosophila) | 10.6 | 6.33E-05 |
| 262 | IL7R | NM_002185 | interleukin 7 receptor | 10.5 | 0.0004685 |
| 263 | PRSS7 | NM_002772 | protease, serine, 7 (enterokinase) | 10.4 | 0.0007137 |
| 264 | ACOX2 | NM_003500 | acyl-Coenzyme A oxidase 2, branched chain | 10.4 | 0.0003963 |
| 265 | CACNA2D4 | AI433691 | calcium channel, voltage-dependent, alpha 2/delta subunit 4 | 10.4 | 3.02E-05 |
| 266 | IFIT3 | NM_001549 | interferon-induced protein with tetratricopeptide repeats 3 | 10.4 | 5.93E-06 |
| 267 | KCTD12 | AA551075 | potassium channel tetramerisation domain containing 12 | 10.3 | 8.74E-05 |
| 268 | GBP1 | NM_002053 | guanylate binding protein 1, interferon-inducible, 67kDa | 10.3 | 3.21E-06 |
| 269 | MCAM | M29277 | melanoma cell adhesion molecule | 10.2 | 0.0002603 |
| 270 | AP1M2 | AA910946 | adaptor-related protein complex 1, mu 2 subunit | 10.2 | 0.0003374 |
| 271 | PCDH7 | NM_002589 | BH-protocadherin (brain-heart) | 10.2 | 2.26E-05 |
| 272 | IBRDC2 | AI953847 | IBR domain containing 2 | 10.2 | 6.51E-06 |
| 273 | KRTAP2-1 | BC012486 | keratin associated protein 2-1 | 10.2 | 2.88E-05 |
| 274 | PDE4D | R40917 | phosphodiesterase 4D, cAMP-specific (phosphodiesterase E3 dunce homolog, Drosophila) | 10.0 | 0.0004997 |
|  |  |  |  |  |  |

*Down-regulation of the genes due to BRCA1 overexpression*

| **Number** | **Symbol** | **GenBank** | **Description** | **Fold** | **raw p-value** |
| --- | --- | --- | --- | --- | --- |
| 1 | SLC16A9 | BG401568 | solute carrier family 16 (monocarboxylic acid transporters), member 9 | -4.0 | 0.00063 |
| 2 | TINAG | BF000045 | tubulointerstitial nephritis antigen | -4.0 | 5.04E-06 |
| 3 | GPM6B | AF016004 | glycoprotein M6B | -4.0 | 0.00048 |
| 4 | RABGAP1L | NM_014857 | RAB GTPase activating protein 1-like | -4.0 | 0.00032 |
| 5 | AOF1 | NM_153042 | amine oxidase (flavin containing) domain 1 | -4.0 | 0.00024 |
| 6 | PARVB | N73272 | parvin, beta | -4.0 | 0.0002 |
| 7 | PPFIBP2 | AI692180 | PTPRF interacting protein, binding protein 2 (liprin beta 2) | -4.1 | 0.00061 |
| 8 | DKK3 | AU144382 | dickkopf homolog 3 (Xenopus laevis) | -4.1 | 7.86E-05 |
| 9 | HOXA7 | AF026397 | homeo box A7 | -4.1 | 4.90E-05 |
| 10 | KCTD15 | NM_024076 | potassium channel tetramerisation domain containing 15 | -4.1 | 6.91E-05 |
| 11 | ATP7B | NM_000053 | ATPase, Cu++ transporting, beta polypeptide (Wilson disease) | -4.1 | 0.00038 |
| 12 | RBPMS | D84109 | RNA binding protein with multiple splicing | -4.1 | 0.00018 |
| 13 | TPM2 | AL566786 | tropomyosin 2 (beta) | -4.1 | 0.00041 |
| 14 | ZNF283 | AI753038 | zinc finger protein 283 | -4.1 | 0.00037 |
| 15 | ATG10 | AL136912 | ATG10 autophagy related 10 homolog (S. cerevisiae) | -4.1 | 3.27E-05 |
| 16 | SESN2 | BF131886 | sestrin 2 | -4.2 | 3.78E-05 |
| 17 | TGIF | NM_003244 | TGFB-induced factor (TALE family homeobox) | -4.2 | 5.34E-05 |
| 18 | C14orf132 | NM_020215 | chromosome 14 open reading frame 132 | -4.2 | 0.00033 |
| 19 | CAMTA1 | Z98884 | calmodulin binding transcription activator 1 | -4.2 | 2.73E-05 |
| 20 | ARRB2 | NM_004313 | arrestin, beta 2 | -4.2 | 0.00015 |
| 21 | ZNF285 | AW513227 | zinc finger protein 285 | -4.2 | 0.00022 |
| 22 | TAF12 | D50544 | TAF12 RNA polymerase II, TATA box binding protein (TBP)-associated factor, 20kDa | -4.2 | 0.00063 |
| 23 | TAF4B | AI366784 | TAF4b RNA polymerase II, TATA box binding protein (TBP)-associated factor, 105kDa | -4.2 | 0.00063 |
| 24 | SPATS2 | NM_023071 | spermatogenesis associated, serine-rich 2 | -4.2 | 1.98E-06 |
| 25 | FDXR | NM_004110 | ferredoxin reductase | -4.2 | 2.08E-05 |
| 26 | SMOX | AY033891 | spermine oxidase | -4.2 | 4.74E-05 |
| 27 | NF1 | D12625 | neurofibromin 1 (neurofibromatosis, von Recklinghausen disease, Watson disease) | -4.3 | 0.00066 |
| 28 | TGIF | AL832409 | TGFB-induced factor (TALE family homeobox) | -4.3 | 0.00015 |
| 29 | CKLFSF3 | AL574900 | chemokine-like factor superfamily 3 | -4.3 | 9.14E-06 |
| 30 | HMGA2 | NM_003483 | high mobility group AT-hook 2 | -4.3 | 1.74E-06 |
| 31 | JAM3 | AF356518 | junctional adhesion molecule 3 | -4.3 | 9.73E-05 |
| 32 | PTPRO | NM_002848 | protein tyrosine phosphatase, receptor type, O | -4.3 | 0.00075 |
| 33 | CIB2 | NM_006383 | calcium and integrin binding family member 2 | -4.3 | 0.00014 |
| 34 | C10orf125 | AI471699 | chromosome 10 open reading frame 125 | -4.3 | 0.0005 |
| 35 | C20orf108 | AI133137 | chromosome 20 open reading frame 108 | -4.4 | 0.00019 |
| 36 | KIAA1598 | AU157109 | KIAA1598 | -4.4 | 2.44E-06 |
| 37 | KIF5C | NM_004522 | kinesin family member 5C | -4.4 | 2.46E-05 |
| 38 | PSG4 | NM_002780 | pregnancy specific beta-1-glycoprotein 4 | -4.4 | 0.00023 |
| 39 | CPEB1 | NM_030594 | cytoplasmic polyadenylation element binding protein 1 | -4.4 | 0.00018 |
| 40 | WDR33 | AW161711 | WD repeat domain 33 | -4.4 | 7.09E-05 |
| 41 | TRIM7 | AA527412 | tripartite motif-containing 7 | -4.5 | 2.83E-05 |
| 42 | ABAT | AF237813 | 4-aminobutyrate aminotransferase | -4.5 | 0.00018 |
| 43 | TUB | AL042088 | tubby homolog (mouse) | -4.5 | 0.00064 |
| 44 | CDCP1 | AK026028 | CUB domain containing protein 1 | -4.5 | 4.20E-06 |
| 45 | GNAS | AA401492 | GNAS complex locus | -4.5 | 0.00058 |
| 46 | OTUB2 | AI656232 | OTU domain, ubiquitin aldehyde binding 2 | -4.6 | 0.00072 |
| 47 | CEBPD | AV655640 | CCAAT/enhancer binding protein (C/EBP), delta | -4.6 | 0.00021 |
| 48 | UCHL1 | NM_004181 | ubiquitin carboxyl-terminal esterase L1 (ubiquitin thiolesterase) | -4.6 | 0.0001 |
| 49 | CPNE8 | AI702381 | copine VIII | -4.6 | 4.14E-05 |
| 50 | ZNF331 | AW450874 | zinc finger protein 331 | -4.6 | 5.48E-05 |
| 51 | PLEKHA1 | NM_021622 | pleckstrin homology domain containing, family A (phosphoinositide binding specific) member 1 | -4.6 | 0.00048 |
| 52 | RBPMS | D84109 | RNA binding protein with multiple splicing | -4.7 | 0.00032 |
| 53 | NFKBIA | AI078167 | nuclear factor of kappa light polypeptide gene enhancer in B-cells inhibitor, alpha | -4.7 | 9.29E-05 |
| 54 | ASRGL1 | AI928342 | asparaginase like 1 | -4.7 | 2.61E-05 |
| 55 | GABRG2 | NM_000816 | gamma-aminobutyric acid (GABA) A receptor, gamma 2 | -4.8 | 0.00015 |
| 56 | SLC19A1 | BQ003811 | solute carrier family 19 (folate transporter), member 1 | -4.8 | 4.64E-06 |
| 57 | MYH10 | AI382123 | myosin, heavy polypeptide 10, non-muscle | -4.8 | 3.81E-05 |
| 58 | ZNF702 | NM_024924 | zinc finger protein 702 | -4.8 | 0.00057 |
| 59 | USP45 | AI091821 | ubiquitin specific peptidase 45 | -4.8 | 0.00044 |
| 60 | BOLA1 | NM_016074 | bolA-like 1 (E. coli) | -4.8 | 9.17E-05 |
| 61 | FUT4 | M58596 | fucosyltransferase 4 (alpha (1,3) fucosyltransferase, myeloid-specific) | -4.8 | 0.00062 |
| 62 | BCHE | NM_000055 | butyrylcholinesterase | -4.9 | 0.00044 |
| 63 | TMEM17 | AA084725 | transmembrane protein 17 | -4.9 | 0.00039 |
| 64 | ZNF599 | AW300140 | zinc finger protein 599 | -4.9 | 1.07E-05 |
| 65 | TFAP2A | BF343007 | transcription factor AP-2 alpha (activating enhancer binding protein 2 alpha) | -4.9 | 8.88E-05 |
| 66 | MMP1 | NM_002421 | matrix metallopeptidase 1 (interstitial collagenase) | -4.9 | 0.00064 |
| 67 | DZIP1 | AL568422 | DAZ interacting protein 1 | -4.9 | 0.00046 |
| 68 | ZNF37B | AI634543 | zinc finger protein 37b (KOX 21) | -4.9 | 0.00053 |
| 69 | OR2A9P | BC040701 | olfactory receptor, family 2, subfamily A, member 9 pseudogene | -5.0 | 0.00052 |
| 70 | TRMT12 | NM_017956 | tRNA methyltranferase 12 homolog (S. cerevisiae) | -5.0 | 3.72E-06 |
| 71 | UBE3A | AL832250 | ubiquitin protein ligase E3A (human papilloma virus E6-associated protein, Angelman syndrome) | -5.0 | 7.02E-05 |
| 72 | FOXE1 | NM_004473 | forkhead box E1 (thyroid transcription factor 2) | -5.0 | 1.46E-05 |
| 73 | C20orf108 | BG432350 | chromosome 20 open reading frame 108 | -5.1 | 2.13E-05 |
| 74 | MAP7 | AW242297 | microtubule-associated protein 7 | -5.1 | 0.00044 |
| 75 | BHLHB3 | BE857425 | basic helix-loop-helix domain containing, class B, 3 | -5.1 | 1.16E-05 |
| 76 | PARVG | AA564926 | parvin, gamma | -5.1 | 9.49E-05 |
| 77 | IRAK2 | AI246590 | interleukin-1 receptor-associated kinase 2 | -5.1 | 1.31E-05 |
| 78 | RAB1A | BG530481 | RAB1A, member RAS oncogene family | -5.1 | 2.92E-05 |
| 79 | CEBPD | NM_005195 | CCAAT/enhancer binding protein (C/EBP), delta | -5.2 | 0.00016 |
| 80 | PCBD1 | NM_000281 | 6-pyruvoyl-tetrahydropterin synthase/dimerization cofactor of hepatocyte nuclear factor 1 alpha (TCF1) | -5.2 | 0.00021 |
| 81 | ZNF558 | AW119060 | zinc finger protein 558 | -5.3 | 0.00031 |
| 82 | TATDN1 | AF212250 | TatD DNase domain containing 1 | -5.3 | 2.98E-06 |
| 83 | FZD8 | AW340311 | frizzled homolog 8 (Drosophila) | -5.3 | 1.02E-07 |
| 84 | DLX2 | NM_004405 | distal-less homeo box 2 | -5.3 | 5.94E-05 |
| 85 | CDKN1A | NM_000389 | cyclin-dependent kinase inhibitor 1A (p21, Cip1) | -5.3 | 1.13E-05 |
| 86 | TRIB3 | NM_021158 | tribbles homolog 3 (Drosophila) | -5.3 | 0.00021 |
| 87 | HOXB7 | S49765 | homeo box B7 | -5.3 | 0.00049 |
| 88 | CAMTA1 | AF111804 | calmodulin binding transcription activator 1 | -5.4 | 0.00014 |
| 89 | ADRB1 | AI625747 | adrenergic, beta-1-, receptor | -5.4 | 7.90E-05 |
| 90 | SIRPB1 | BC025286 | signal-regulatory protein beta 1 | -5.4 | 6.17E-05 |
| 91 | DDB2 | NM_000107 | damage-specific DNA binding protein 2, 48kDa | -5.4 | 7.01E-05 |
| 92 | TRIB3 | AF250311 | tribbles homolog 3 (Drosophila) | -5.5 | 2.20E-05 |
| 93 | CLYBL | BG398847 | citrate lyase beta like | -5.5 | 7.31E-05 |
| 94 | HMOX1 | NM_002133 | heme oxygenase (decycling) 1 | -5.5 | 7.97E-06 |
| 95 | TLE4 | AL358975 | transducin-like enhancer of split 4 (E(sp1) homolog, Drosophila) | -5.5 | 0.00035 |
| 96 | SPATS2 | AA584308 | spermatogenesis associated, serine-rich 2 | -5.5 | 0.00028 |
| 97 | KLHL13 | AB037730 | kelch-like 13 (Drosophila) | -5.6 | 0.00028 |
| 98 | ELF3 | U73844 | E74-like factor 3 (ets domain transcription factor, epithelial-specific ) | -5.6 | 3.64E-06 |
| 99 | GABRG2 | BC036030 | gamma-aminobutyric acid (GABA) A receptor, gamma 2 | -5.7 | 0.00015 |
| 100 | ICAM1 | AA284705 | intercellular adhesion molecule 1 (CD54), human rhinovirus receptor | -5.7 | 0.00021 |
| 101 | KDELC1 | NM_024089 | KDEL (Lys-Asp-Glu-Leu) containing 1 | -5.8 | 0.00014 |
| 102 | ELF3 | AF017307 | E74-like factor 3 (ets domain transcription factor, epithelial-specific ) | -5.8 | 0.00012 |
| 103 | SOX9 | AI382146 | SRY (sex determining region Y)-box 9 (campomelic dysplasia, autosomal sex-reversal) | -5.8 | 3.27E-05 |
| 104 | CKLFSF3 | AY166714 | chemokine-like factor superfamily 3 | -5.9 | 0.00031 |
| 105 | CLMN | AW028110 | calmin (calponin-like, transmembrane) | -6.0 | 5.06E-06 |
| 106 | BMP7 | M60316 | bone morphogenetic protein 7 (osteogenic protein 1) | -6.1 | 4.99E-05 |
| 107 | KIAA1411 | AL136820 | KIAA1411 | -6.1 | 1.68E-06 |
| 108 | IL13RA2 | NM_000640 | interleukin 13 receptor, alpha 2 | -6.1 | 0.00025 |
| 109 | DLL3 | NM_016941 | delta-like 3 (Drosophila) | -6.1 | 0.00014 |
| 110 | MSX1 | NM_002448 | msh homeo box homolog 1 (Drosophila) | -6.2 | 0.00014 |
| 111 | ZNF677 | AK026366 | zinc finger protein 677 | -6.2 | 0.00012 |
| 112 | ASRGL1 | NM_025080 | asparaginase like 1 | -6.2 | 0.00035 |
| 113 | PITPNC1 | NM_012417 | phosphatidylinositol transfer protein, cytoplasmic 1 | -6.2 | 0.00059 |
| 114 | PLCXD1 | NM_018390 | phosphatidylinositol-specific phospholipase C, X domain containing 1 | -6.2 | 5.97E-05 |
| 115 | PADI2 | BC009701 | peptidyl arginine deiminase, type II | -6.2 | 0.00014 |
| 116 | XK | NM_021083 | Kell blood group precursor (McLeod phenotype) | -6.2 | 0.00017 |
| 117 | NF2 | AF123570 | neurofibromin 2 (bilateral acoustic neuroma) | -6.3 | 2.26E-05 |
| 118 | PCDHB6 | AI821557 | protocadherin beta 6 | -6.3 | 0.00038 |
| 119 | PRKD1 | NM_002742 | protein kinase D1 | -6.3 | 3.02E-05 |
| 120 | HPS3 | AI922198 | Hermansky-Pudlak syndrome 3 | -6.3 | 1.82E-05 |
| 121 | PAX6 | NM_000280 | paired box gene 6 (aniridia, keratitis) | -6.4 | 1.16E-05 |
| 122 | ZNF239 | NM_005674 | zinc finger protein 239 | -6.4 | 0.00076 |
| 123 | NF2 | NM_016418 | neurofibromin 2 (bilateral acoustic neuroma) | -6.5 | 4.85E-06 |
| 124 | NNMT | NM_006169 | nicotinamide N-methyltransferase | -6.5 | 0.00022 |
| 125 | ZNF505 | NM_031218 | zinc finger protein 505 | -6.6 | 2.51E-05 |
| 126 | HORMAD1 | AL136755 | HORMA domain containing 1 | -6.6 | 6.69E-05 |
| 127 | HOXA5 | NM_019102 | homeo box A5 | -6.6 | 8.71E-05 |
| 128 | NID1 | BF940043 | nidogen 1 | -6.6 | 6.74E-07 |
| 129 | C20orf133 | BC035876 | chromosome 20 open reading frame 133 | -6.7 | 3.98E-06 |
| 130 | TMLHE | AI635160 | trimethyllysine hydroxylase, epsilon | -6.7 | 0.00074 |
| 131 | ZNF232 | AI693543 | zinc finger protein 232 | -6.7 | 0.00013 |
| 132 | NF2 | AF122828 | neurofibromin 2 (bilateral acoustic neuroma) | -6.7 | 0.00033 |
| 133 | PAX8 | AU154891 | paired box gene 8 | -6.7 | 0.00067 |
| 134 | NNMT | NM_006169 | nicotinamide N-methyltransferase | -6.8 | 0.00025 |
| 135 | FGF2 | NM_002006 | fibroblast growth factor 2 (basic) | -6.8 | 0.00014 |
| 136 | NRIP3 | NM_020645 | nuclear receptor interacting protein 3 | -6.8 | 0.00027 |
| 137 | HOXA9 | U41813 | homeo box A9 | -6.9 | 2.11E-05 |
| 138 | LDOC1 | NM_012317 | leucine zipper, down-regulated in cancer 1 | -7.0 | 7.12E-05 |
| 139 | ZNF677 | AI816281 | zinc finger protein 677 | -7.0 | 3.14E-06 |
| 140 | CASK | AI659225 | calcium/calmodulin-dependent serine protein kinase (MAGUK family) | -7.1 | 0.00022 |
| 141 | RAB3B | BC005035 | RAB3B, member RAS oncogene family | -7.2 | 0.00047 |
| 142 | PCDHA6 | AI268404 | protocadherin alpha 6 | -7.2 | 1.70E-06 |
| 143 | SMOX | BC000669 | spermine oxidase | -7.2 | 2.26E-05 |
| 144 | ZNF256 | BC001438 | zinc finger protein 256 | -7.2 | 0.00065 |
| 145 | PTPNS1 | NM_004648 | protein tyrosine phosphatase, non-receptor type substrate 1 | -7.3 | 0.00016 |
| 146 | C1orf115 | NM_024709 | chromosome 1 open reading frame 115 | -7.4 | 0.00032 |
| 147 | TMEPAI | AL035541 | transmembrane, prostate androgen induced RNA | -7.4 | 0.00057 |
| 148 | PPP2R2C | AI669212 | protein phosphatase 2 (formerly 2A), regulatory subunit B (PR 52), gamma isoform | -7.5 | 1.99E-05 |
| 149 | HOXB3 | AW510657 | homeo box B3 | -7.5 | 0.00052 |
| 150 | RFP2 | BF939833 | ret finger protein 2 | -7.5 | 0.00066 |
| 151 | GPR51 | AF056085 | G protein-coupled receptor 51 | -7.5 | 0.00066 |
| 152 | TGM2 | AL031651 | transglutaminase 2 (C polypeptide, protein-glutamine-gamma-glutamyltransferase) | -7.5 | 2.36E-05 |
| 153 | CP | NM_000096 | ceruloplasmin (ferroxidase) | -7.6 | 0.00044 |
| 154 | PLK2 | NM_006622 | polo-like kinase 2 (Drosophila) | -7.6 | 8.25E-06 |
| 155 | PCDHGC3 | NM_002588 | protocadherin gamma subfamily C, 3 | -7.6 | 0.00036 |
| 156 | SLC16A1 | BF511091 | solute carrier family 16 (monocarboxylic acid transporters), member 1 | -7.8 | 5.26E-06 |
| 157 | PCDHGC3 | BC006439 | protocadherin gamma subfamily C, 3 | -7.9 | 7.60E-05 |
| 158 | JAM3 | AA149644 | junctional adhesion molecule 3 | -7.9 | 2.64E-05 |
| 159 | FZD8 | AB043703 | frizzled homolog 8 (Drosophila) | -8.0 | 0.00029 |
| 160 | ARHGAP8 | Z83838 | Rho GTPase activating protein 8 | -8.0 | 9.92E-05 |
| 161 | ZNF329 | NM_024620 | zinc finger protein 329 | -8.0 | 1.46E-06 |
| 162 | DCBLD2 | AI378788 | discoidin, CUB and LCCL domain containing 2 | -8.0 | 0.00034 |
| 163 | CRIP1 | NM_001311 | cysteine-rich protein 1 (intestinal) | -8.1 | 0.00058 |
| 164 | SNRPN | BG413612 | small nuclear ribonucleoprotein polypeptide N | -8.1 | 0.00065 |
| 165 | GRB14 | NM_004490 | growth factor receptor-bound protein 14 | -8.1 | 7.11E-05 |
| 166 | PCDHGC3 | AF152524 | protocadherin gamma subfamily C, 3 | -8.3 | 8.24E-05 |
| 167 | TMEM47 | AL136550 | transmembrane protein 47 | -8.4 | 9.44E-06 |
| 168 | PAPLN | AU145309 | papilin, proteoglycan-like sulfated glycoprotein | -8.5 | 6.60E-05 |
| 169 | PARVB | NM_013327 | parvin, beta | -8.5 | 0.00054 |
| 170 | HOXA1 | S79910 | homeo box A1 | -8.6 | 0.00011 |
| 171 | HOXB7 | NM_004502 | homeo box B7 | -8.6 | 0.00024 |
| 172 | SLC35F5 | NM_025181 | solute carrier family 35, member F5 | -8.8 | 3.79E-05 |
| 173 | TNF | NM_000594 | tumor necrosis factor (TNF superfamily, member 2) | -8.8 | 0.00018 |
| 174 | CSPG2 | R94644 | chondroitin sulfate proteoglycan 2 (versican) | -8.9 | 0.0001 |
| 175 | EFNB3 | NM_001406 | ephrin-B3 | -8.9 | 8.19E-07 |
| 176 | HDAC9 | NM_014707 | histone deacetylase 9 | -9.0 | 8.13E-05 |
| 177 | CSPG2 | NM_004385 | chondroitin sulfate proteoglycan 2 (versican) | -9.3 | 1.25E-06 |
| 178 | RGS17 | NM_012419 | regulator of G-protein signalling 17 | -9.4 | 0.00028 |
| 179 | HAVCR1 | NM_012206 | hepatitis A virus cellular receptor 1 | -9.6 | 3.67E-05 |
| 180 | RASGEF1A | BF446578 | RasGEF domain family, member 1A | -9.7 | 0.00028 |
| 181 | CD109 | AL110152 | CD109 antigen (Gov platelet alloantigens) | -9.7 | 9.03E-05 |
| 182 | EBI3 | NM_005755 | Epstein-Barr virus induced gene 3 | -9.7 | 0.0007 |
| 183 | LHX2 | NM_004789 | LIM homeobox 2 | -10.0 | 3.20E-06 |
| 184 | DDX43 | NM_018665 | DEAD (Asp-Glu-Ala-Asp) box polypeptide 43 | -10.1 | 0.00025 |
| 185 | TRIM7 | AF220032 | tripartite motif-containing 7 | -10.6 | 3.07E-06 |
| 186 | VCAM1 | NM_001078 | vascular cell adhesion molecule 1 | -10.9 | 3.44E-05 |
| 187 | KIAA1622 | AB046842 | KIAA1622 | -11.0 | 0.00039 |
| 188 | LTBP2 | NM_000428 | latent transforming growth factor beta binding protein 2 | -11.1 | 5.82E-05 |
| 189 | FKBP1B | NM_004116 | FK506 binding protein 1B, 12.6 kDa | -11.2 | 0.00014 |
| 190 | GSPT2 | NM_018094 | G1 to S phase transition 2 | -11.3 | 0.00039 |
| 191 | RNF144 | NM_014746 | ring finger protein 144 | -11.3 | 6.61E-05 |
| 192 | PAX6 | AW088232 | paired box gene 6 (aniridia, keratitis) | -11.5 | 0.00029 |
| 193 | KCNJ16 | NM_018658 | potassium inwardly-rectifying channel, subfamily J, member 16 | -11.7 | 1.70E-05 |
| 194 | CXCL6 | NM_002993 | chemokine (C-X-C motif) ligand 6 (granulocyte chemotactic protein 2) | -11.7 | 0.00029 |
| 195 | SLC19A1 | BC003068 | solute carrier family 19 (folate transporter), member 1 | -12.0 | 1.12E-05 |
| 196 | EFNA1 | NM_004428 | ephrin-A1 | -12.0 | 0.00012 |
| 197 | TGM2 | BC003551 | transglutaminase 2 (C polypeptide, protein-glutamine-gamma-glutamyltransferase) | -12.1 | 0.00029 |
| 198 | SOD2 | X15132 | superoxide dismutase 2, mitochondrial | -12.1 | 0.00067 |
| 199 | GJA7 | BE504215 | gap junction protein, alpha 7, 45kDa (connexin 45) | -12.2 | 3.66E-05 |
| 200 | CSPG2 | BF218922 | chondroitin sulfate proteoglycan 2 (versican) | -12.4 | 9.81E-05 |
| 201 | SAA2 | M23699 | serum amyloid A2 | -12.4 | 0.00036 |
| 202 | TGM2 | M98478 | transglutaminase 2 (C polypeptide, protein-glutamine-gamma-glutamyltransferase) | -12.8 | 1.71E-05 |
| 203 | GAL | AL556409 | galanin | -12.8 | 1.42E-05 |
| 204 | TNFAIP3 | AI738896 | tumor necrosis factor, alpha-induced protein 3 | -12.9 | 4.30E-06 |
| 205 | HIST1H2BG | BC001131 | histone 1, H2bg | -13.1 | 5.82E-05 |
| 206 | CLDN16 | NM_006580 | claudin 16 | -13.2 | 2.28E-05 |
| 207 | CP | AL556703 | ceruloplasmin (ferroxidase) | -13.2 | 1.29E-05 |
| 208 | RGS20 | AF074979 | regulator of G-protein signalling 20 | -13.3 | 0.0002 |
| 209 | PARVB | AA187563 | parvin, beta | -13.3 | 3.16E-05 |
| 210 | QPRT | AW960707 | quinolinate phosphoribosyltransferase (nicotinate-nucleotide pyrophosphorylase (carboxylating)) | -13.6 | 6.40E-05 |
| 211 | LCN2 | NM_005564 | lipocalin 2 (oncogene 24p3) | -13.7 | 0.00013 |
| 212 | CCND2 | AW026491 | cyclin D2 | -13.9 | 6.85E-06 |
| 213 | HOXA3 | AW137982 | homeo box A3 | -14.0 | 2.85E-05 |
| 214 | CDKN2A | U38945 | cyclin-dependent kinase inhibitor 2A (melanoma, p16, inhibits CDK4) | -14.2 | 1.11E-06 |
| 215 | PTPRO | BC035960 | protein tyrosine phosphatase, receptor type, O | -14.7 | 6.69E-07 |
| 216 | NFKBIZ | AB037925 | nuclear factor of kappa light polypeptide gene enhancer in B-cells inhibitor, zeta | -14.8 | 0.00023 |
| 217 | TNFAIP6 | NM_007115 | tumor necrosis factor, alpha-induced protein 6 | -14.9 | 2.03E-06 |
| 218 | CXCL2 | M57731 | chemokine (C-X-C motif) ligand 2 | -15.3 | 0.00041 |
| 219 | TPM1 | NM_000366 | tropomyosin 1 (alpha) | -15.4 | 0.00028 |
| 220 | QPRT | NM_014298 | quinolinate phosphoribosyltransferase (nicotinate-nucleotide pyrophosphorylase (carboxylating)) | -15.5 | 0.00023 |
| 221 | TEX15 | AL133653 | testis expressed sequence 15 | -15.6 | 1.13E-06 |
| 222 | DCDC2 | AW444617 | doublecortin domain containing 2 | -15.6 | 0.00075 |
| 223 | KIAA1797 | NM_017794 | KIAA1797 | -15.8 | 0.00043 |
| 224 | IL8 | NM_000584 | interleukin 8 | -16.4 | 6.01E-05 |
| 225 | CLDN1 | AI924046 | claudin 1 | -17.0 | 0.00075 |
| 226 | SOD2 | W46388 | superoxide dismutase 2, mitochondrial | -17.3 | 0.00016 |
| 227 | PRAME | NM_006115 | preferentially expressed antigen in melanoma | -17.6 | 3.76E-05 |
| 228 | COCH | BC007230 | coagulation factor C homolog, cochlin (Limulus polyphemus) | -17.6 | 1.86E-06 |
| 229 | ICAM1 | AI608725 | intercellular adhesion molecule 1 (CD54), human rhinovirus receptor | -17.7 | 0.00013 |
| 230 | NFKBIZ | BE646573 | nuclear factor of kappa light polypeptide gene enhancer in B-cells inhibitor, zeta | -17.7 | 7.60E-07 |
| 231 | CLDN11 | AW264204 | claudin 11 (oligodendrocyte transmembrane protein) | -18.6 | 6.88E-07 |
| 232 | RAB3B | AU156710 | RAB3B, member RAS oncogene family | -19.5 | 1.16E-05 |
| 233 | KCTD15 | W73820 | potassium channel tetramerisation domain containing 15 | -20.1 | 0.00022 |
| 234 | PTPNS1 | AB023430 | protein tyrosine phosphatase, non-receptor type substrate 1 | -20.1 | 0.00073 |
| 235 | SLC16A1 | BF511091 | solute carrier family 16 (monocarboxylic acid transporters), member 1 | -20.9 | 8.85E-06 |
| 236 | CDKN2A | NM_000077 | cyclin-dependent kinase inhibitor 2A (melanoma, p16, inhibits CDK4) | -21.4 | 5.64E-05 |
| 237 | PAX8 | NM_013992 | paired box gene 8 | -21.5 | 6.33E-05 |
| 238 | COCH | AA669336 | coagulation factor C homolog, cochlin (Limulus polyphemus) | -21.6 | 0.00059 |
| 239 | SLCO4A1 | NM_016354 | solute carrier organic anion transporter family, member 4A1 | -22.1 | 9.46E-05 |
| 240 | CLDN1 | AF101051 | claudin 1 | -22.5 | 9.80E-07 |
| 241 | ICAM1 | NM_000201 | intercellular adhesion molecule 1 (CD54), human rhinovirus receptor | -22.8 | 0.00019 |
| 242 | EIF5A | BC000751 | eukaryotic translation initiation factor 5A | -23.1 | 5.04E-05 |
| 243 | TKTL1 | X91817 | transketolase-like 1 | -23.3 | 2.67E-05 |
| 244 | SNRPN | BE783065 | small nuclear ribonucleoprotein polypeptide N | -23.8 | 3.52E-05 |
| 245 | AMFR | NM_001144 | autocrine motility factor receptor | -24.6 | 9.06E-07 |
| 246 | LTBP2 | AF113211 | latent transforming growth factor beta binding protein 2 | -26.7 | 0.00042 |
| 247 | PAX8 | NM_013951 | paired box gene 8 | -29.0 | 0.00025 |
| 248 | GDF15 | AF003934 | growth differentiation factor 15 | -29.6 | 0.00013 |
| 249 | KCNJ2 | BF111326 | potassium inwardly-rectifying channel, subfamily J, member 2 | -30.1 | 0.00021 |
| 250 | KLHL9 | AA460694 | kelch-like 9 (Drosophila) | -30.8 | 0.00052 |
| 251 | IAPP | NM_000415 | islet amyloid polypeptide | -31.6 | 6.32E-05 |
| 252 | SNRPN | BF114870 | small nuclear ribonucleoprotein polypeptide N | -32.9 | 8.85E-08 |
| 253 | CBS | BC007257 | cystathionine-beta-synthase | -34.5 | 5.70E-05 |
| 254 | DKK3 | AU148057 | dickkopf homolog 3 (Xenopus laevis) | -36.2 | 3.71E-06 |
| 255 | C1orf59 | BE502436 | chromosome 1 open reading frame 59 | -36.8 | 6.85E-05 |
| 256 | C14orf105 | NM_018168 | chromosome 14 open reading frame 105 | -37.1 | 5.09E-06 |
| 257 | BNC1 | NM_001717 | basonuclin 1 | -44.2 | 8.43E-07 |
| 258 | CBS | BE613178 | cystathionine-beta-synthase | -46.3 | 2.46E-05 |
| 259 | STMN3 | AL353715 | stathmin-like 3 | -46.7 | 9.41E-05 |
| 260 | SLC16A1 | AL162079 | solute carrier family 16 (monocarboxylic acid transporters), member 1 | -49.2 | 1.76E-06 |
| 261 | COL5A2 | AL575735 | collagen, type V, alpha 2 | -49.9 | 0.00058 |
| 262 | DMD | NM_004010 | dystrophin (muscular dystrophy, Duchenne and Becker types) | -52.9 | 3.15E-05 |
| 263 | SLC16A1 | NM_003051 | solute carrier family 16 (monocarboxylic acid transporters), member 1 | -56.3 | 9.45E-05 |
| 264 | BIRC3 | U37546 | baculoviral IAP repeat-containing 3 | -57.4 | 0.00012 |
| 265 | EIF5A | NM_001970 | eukaryotic translation initiation factor 5A | -58.5 | 1.11E-06 |
| 266 | PDZK1IP1 | NM_005764 | PDZK1 interacting protein 1 | -60.6 | 6.97E-05 |
| 267 | VNN1 | BG120535 | vanin 1 | -68.1 | 0.00052 |
| 268 | NETO2 | NM_018092 | neuropilin (NRP) and tolloid (TLL)-like 2 | -69.1 | 5.98E-05 |
| 269 | RPS4Y1 | NM_001008 | ribosomal protein S4, Y-linked 1 | -71.7 | 0.00017 |
| 270 | DKK3 | AL569601 | dickkopf homolog 3 (Xenopus laevis) | -77.8 | 3.83E-07 |
| 271 | COL5A2 | NM_000393 | collagen, type V, alpha 2 | -79.0 | 0.00015 |
| 272 | NETO2 | AI335263 | neuropilin (NRP) and tolloid (TLL)-like 2 | -85.9 | 5.61E-06 |
| 273 | HSPA1A | NM_005345 | heat shock 70kDa protein 1A | -93.7 | 0.00025 |
| 274 | GJA7 | AA430014 | gap junction protein, alpha 7, 45kDa (connexin 45) | -116.8 | 1.96E-05 |
| 275 | CCND2 | NM_001759 | cyclin D2 | -122.5 | 4.86E-08 |
| 276 | HAS3 | AF232772 | hyaluronan synthase 3 | -128.3 | 0.00024 |
| 277 | C3 | NM_000064 | complement component 3 | -130.2 | 5.26E-05 |
| 278 | FEZ1 | NM_005103 | fasciculation and elongation protein zeta 1 (zygin I) | -281.4 | 0.00074 |
| 279 | VNN1 | NM_004666 | vanin 1 | -321.4 | 3.09E-07 |
