# Supplementary material for: BRCA1 Regulates Follistatin Function in Ovarian Cancer and Human Ovarian Surface Epithelial Cells
Source: PLoS One. 2012 Jun 1;7(6):e37697. doi: 10.1371/journal.pone.0037697 (PMC3365892; doi:10.1371/journal.pone.0037697)
Supplement: Table S2 — Pathway specific up-regulation of the genes with the up-regulation of BRCA1 in SKOV3 cells. (DOC) [file pone.0037697.s004.doc]

| **Pathways** | **Accession number** | **Gene name** | **Symbol** | **Folds Change** | **p-value** |
| --- | --- | --- | --- | --- | --- |
|  |  |  |  |  |  |
| **Glycolysis / Gluconeogenesis** | | |  |  |  |
|  | BE788984 | galactose mutarotase (aldose 1-epimerase) | GALM | 3.8 | 0.00001 |
|  | NM_000694 | aldehyde dehydrogenase 3 family, member B1 | ALDH3B1 | 4.0 | 0.00051 |
|  | NM_005165  **Table S2: Pathway specific up-regulation of the genes with the up-regulation of BRCA1 in SKOV3 cells** | aldolase C, fructose-bisphosphate | ALDOC | 11.6 | 0.00004 |
| **Fatty acid metabolism** | |  |  |  |  |
|  | AL525798 | acyl-CoA synthetase long-chain family member 3 | ACSL3 | 3.2 | 0.00073 |
|  | AU144855 | cytochrome P450, family 1, subfamily B, polypeptide 1 | CYP1B1 | 3.3 | 0.00021 |
|  | NM_003500 | acyl-Coenzyme A oxidase 2, branched chain | ACOX2 | 10.4 | 0.00040 |
|  | NM_016234 | acyl-CoA synthetase long-chain family member 5 | ACSL5 | 25.6 | 0.00001 |
| **Amino acids metabolism** | |  |  |  |  |
|  | NM_002318 | lysyl oxidase-like 2 | LOXL2 | 3.3 | 0.00012 |
|  | NM_000480 | adenosine monophosphate deaminase (isoform E) | AMPD3 | 3.4 | 0.00026 |
|  | AK024946 | dihydrolipoamide branched chain transacylase E2 | DBT | 3.8 | 0.00036 |
|  | M55580 | spermidine/spermine N1-acetyltransferase | SAT | 4.4 | 0.00015 |
|  | NM_002413 | microsomal glutathione S-transferase 2 | MGST2 | 4.8 | 0.00006 |
|  | BC006230 | monoglyceride lipase | MGLL | 6 | 0.00008 |
|  | R40917 | phosphodiesterase 4D | PDE4D | 10 | 0.0005 |
|  | NM_006033 | lipase, endothelial | LIPG | 11.3 | 0.00001 |
|  | NM_001159 | aldehyde oxidase 1 | AOX1 | 12.2 | 0.0001 |
|  | AL527430 | glutathione S-transferase M3 | GSTM3 | 15.2 | 0.00025 |
|  | W93728 | guanylate cyclase 1, soluble, beta 3 | GUCY1B3 | 16.1 | 0.00013 |
|  | NM_005576 | lysyl oxidase-like 1 | LOXL1 | 17.3 | 0.00004 |
|  | NM_002084 | glutathione peroxidase 3 (plasma) | GPX3 | 18.7 | 0.00006 |
|  | D16947 | microsomal glutathione S-transferase 1 | MGST1 | 23.1 | 0.0001 |
|  | NM_002600 | phosphodiesterase 4B | PDE4B | 24.2 | 0.00008 |
|  | AW292746 | myosin regulatory light chain interacting protein | MYLIP | 52.6 | 0.00004 |
| **Phosphatidylinositol signaling system** | | |  |  |  |
|  | NM_002194 | inositol polyphosphate-1-phosphatase | INPP1 | 3.2 | 0.00039 |
|  | NM_016341 | phospholipase C, epsilon 1 | PLCE1 | 5.0 | 0.00024 |
|  | BF218115 | homeodomain interacting protein kinase 2 | HIPK2 | 8.3 | 0.00001 |
|  | AL049593 | phospholipase C, beta 1 (phosphoinositide-specific) | PLCB1 | 18.8 | 0.00006 |
| **Glycosylphosphatidylinositol(GPI)-anchor biosynthesis** | | | |  |  |
|  | AU144243 | phosphatidylinositol glycan, class B | PIGB | 3.1 | 0.00003 |
|  | AA613031 | glycerol-3-phosphate dehydrogenase 2 (mitochondrial) | GPD2 | 4.8 | 0.00035 |
| **Prostaglandin and leukotriene metabolism** | | |  |  |  |
|  | NM_001236 | carbonyl reductase 3 | CBR3 | 4.0 | 0.00001 |
|  | AF010316 | prostaglandin E synthase | PTGES | 5.4 | 0.00045 |
|  | AB018580 | aldo-keto reductase family 1, member C3 | AKR1C3 | 27.4 | 0.00018 |
| **ABC transporters** | |  |  |  |  |
|  | NM_005689 | ATP-binding cassette, sub-family B (MDR/TAP), member 6 | ABCB6 | 3.0 | 0.00003 |
|  | AI084637 | ATP-binding cassette, sub-family C (CFTR/MRP), member 6 | ABCC6 | 5.9 | 0.00019 |
|  | NM_020037 | ATP-binding cassette, sub-family C (CFTR/MRP), member 3 | ABCC3 | 8.7 | 0.00001 |
|  | NM_007168 | ATP-binding cassette, sub-family A (ABC1), member 8 | ABCA8 | 19.6 | 0.00003 |
| **Cell cycle** |  |  |  |  |  |
|  | NM_001262 | cyclin-dependent kinase inhibitor 2C (p18, inhibits CDK4) | CDKN2C | 4.3 | 0.00027 |
|  | NM_003914 | cyclin A1 | CCNA1 | 16.3 | 0.00023 |
| **Notch signaling pathway** | |  |  |  |  |
|  | AI769569 | mastermind-like 2 (Drosophila) | MAML2 | 5.1 | 0.00016 |
|  | U77914 | jagged 1 (Alagille syndrome) | JAG1 | 42.1 | 0.00012 |
| **MAPK signaling pathway** | |  |  |  |  |
|  | AB016517 | fibroblast growth factor 5 | FGF5 | 3.8 | 0.00027 |
|  | NM_001540 | heat shock 27kDa protein 1 | HSPB1 | 3.9 | 0.00006 |
|  | AK000106 | epidermal growth factor receptor | EGFR | 4.1 | 0.00004 |
|  | NM_004417 | dual specificity phosphatase 1 | DUSP1 | 4.8 | 0.00001 |
|  | BC003143 | dual specificity phosphatase 6 | DUSP6 | 5.0 | 0.00000 |
|  | AI770171 | nuclear factor of activated T-cells, calcineurin-dependent 2 | NFATC2 | 6.4 | 0.00022 |
|  | BC000433 | mitogen-activated protein kinase 13 | MAPK13 | 6.9 | 0.00012 |
|  | N22468 | MADS box transcription enhancer factor 2, polypeptide C | MEF2C | 7.0 | 0.00062 |
|  | AI471375 | protein kinase C, alpha | PRKCA | 16.0 | 0.00000 |
|  | BE138888 | ras-related C3 botulinum toxin substrate 2 | RAC2 | 41.0 | 0.00010 |
|  | M19154 | transforming growth factor, beta 2 | TGFB2 | 70.8 | 0.00000 |
|  | NM_004114 | fibroblast growth factor 13 | FGF13 | 71.8 | 0.00000 |
|  | NM_001394 | dual specificity phosphatase 4 | DUSP4 | 255.1 | 0.00000 |
| **Calcium signaling pathway** | |  |  |  |  |
|  | AW576457 | ATPase, Ca++ transporting, plasma membrane 1 | ATP2B1 | 3.0 | 0.00026 |
|  | NM_000861 | histamine receptor H1 | HRH1 | 3.1 | 0.00030 |
|  | AA526844 | myosin, light polypeptide kinase | MYLK | 3.3 | 0.00015 |
|  | AL529104 | calcium/calmodulin-dependent protein kinase IV | CAMK4 | 4.1 | 0.00003 |
|  | NM_000024 | adrenergic, beta-2-, receptor, surface | ADRB2 | 4.2 | 0.00001 |
|  | BF797381 | calcium/calmodulin-dependent protein kinase II delta | CAMK2D | 5.7 | 0.00004 |
|  | AK056349 | cholinergic receptor, muscarinic 3 | CHRM3 | 6.1 | 0.00027 |
|  | NM_001957 | endothelin receptor type A | EDNRA | 7.1 | 0.00006 |
| **Wnt signaling pathway** | |  |  |  |  |
|  | AI263909 | ras homolog gene family, member B | RHOB | 3.2 | 0.00052 |
|  | AV753028 | transducin (beta)-like 1X-linked | TBL1X | 3.5 | 0.00067 |
|  | AA890373 | dishevelled associated activator of morphogenesis 1 | DAAM1 | 4.0 | 0.00040 |
|  | AA463626 | wingless-type MMTV integration site family, member 3 | WNT3 | 4.3 | 0.00064 |
|  | AF288571 | lymphoid enhancer-binding factor 1 | LEF1 | 4.9 | 0.00066 |
|  | BF513244 | dishevelled associated activator of morphogenesis 2 | DAAM2 | 5.0 | 0.00001 |
|  | AI333651 | frizzled homolog 7 (Drosophila) | FZD7 | 5.2 | 0.00012 |
|  | NM_003505 | frizzled homolog 1 (Drosophila) | FZD1 | 5.6 | 0.00006 |
|  | AI569872 | frizzled homolog 4 (Drosophila) | FZD4 | 6.8 | 0.00002 |
|  | NM_003392 | wingless-type MMTV integration site family, member 5A | WNT5A | 60.9 | 0.00002 |
| **TGF-β signaling pathway** | | |  |  |  |
|  | AL022726 | inhibitor of DNA binding 4, | ID4 | 6.4 | 0.00005 |
|  | NM_005585 | SMAD, mothers against DPP homolog 6 (Drosophila) | SMAD6 | 10.7 | 0.00000 |
|  | AV700621 | transforming growth factor, beta receptor I (activin A receptor type II-like kinase, 53kDa) | TGFBR1 | 3.9 | 0.00020 |
|  | M19154 | transforming growth factor, beta 2 | TGFB2 | 70.8 | 0.00000 |
|  | BF438173 | follistatin | FST | 51.6 | 0.00002 |
| **Toll-like receptor signaling pathway** | | |  |  |  |
|  | AI423165 | toll-like receptor adaptor molecule 2 | TICAM2 | 5.3 | 0.00000 |
|  | NM_015364 | lymphocyte antigen 96 | LY96 | 12.0 | 0.00000 |
| **Jak-STAT signaling pathway** | | |  |  |  |
|  | AK024556 | sprouty homolog 4 (Drosophila) | SPRY4 | 3.8 | 0.00002 |
| **Hematopoietic cell lineage** | |  |  |  |  |
|  | BG327863 | CD24 antigen (small cell lung carcinoma cluster 4 antigen) | CD24 | 4.6 | 0.00027 |
|  |  |  |  |  |  |
|  |  |  |  |  |  |
|  |  |  |  |  |  |
| **Focal adhesion** | |  |  |  |  |
|  | BF000430 | actinin, alpha 4 | ACTN4 | 3.0 | 0.00051 |
|  | AF130095 | fibronectin 1 | FN1 | 3.2 | 0.00017 |
|  | AL044092 | insulin-like growth factor 1 receptor | IGF1R | 3.9 | 0.00002 |
|  | AI130969 | collagen, type V, alpha 1 | COL5A1 | 4.6 | 0.00018 |
|  | M14333 | FYN oncogene related to SRC, FGR, YES | FYN | 5.3 | 0.00004 |
|  | L25541 | laminin, beta 3 | LAMB3 | 8.1 | 0.00027 |
|  | NM_025208 | platelet derived growth factor D | PDGFD | 12.1 | 0.00018 |
|  | N95414 | integrin, alpha 2 (CD49B, alpha 2 subunit of VLA-2 receptor) | ITGA2 | 12.1 | 0.00012 |
|  | BC001422 | placental growth factor, VEGF related protein | PGF | 20.1 | 0.00007 |
| **Cell adhesion molecules (CAMs)** | | |  |  |  |
|  | NM_024883 | cadherin 4, type 1, R-cadherin (retinal) | CDH4 | 3.1 | 0.00006 |
|  | NM_001305 | claudin 4 | CLDN4 | 4.1 | 0.00008 |
|  | AL573851 | endothelial cell adhesion molecule | ESAM | 5.3 | 0.00031 |
|  | NM_014932 | neuroligin 1 | NLGN1 | 6.7 | 0.00042 |
|  | AA156721 | activated leukocyte cell adhesion molecule | ALCAM | 8.1 | 0.00000 |
|  | AI608902 | CD274 antigen | CD274 | 17.4 | 0.00025 |
|  | AL577322 | syndecan 2 (heparan sulfate proteoglycan 1 | SDC2 | 21.1 | 0.00002 |
| **Complement and coagulation cascades** | | |  |  |  |
|  | AY029180 | plasminogen activator, urokinase receptor | PLAUR | 4.8 | 0.00004 |
|  | NM_000129 | coagulation factor XIII, A1 polypeptide | F13A1 | 8.3 | 0.00066 |
|  | AL574210 | serpin peptidase inhibitor, clade E (nexin, plasminogen activator inhibitor type 1), member 1 | SERPINE1 | 8.9 | 0.00007 |
|  | NM_000361 | thrombomodulin | THBD | 10.8 | 0.00044 |
|  | X04697 | complement factor H | CFH | 11.4 | 0.00003 |
|  | NM_000716 | complement component 4 binding protein, beta | C4BPB | 13.5 | 0.00039 |
|  | NM_000930 | plasminogen activator, tissue | PLAT | 20.7 | 0.00002 |
|  | NM_001993 | coagulation factor III (thromboplastin, tissue factor) | F3 | 33.6 | 0.00020 |
| **Cytokine-cytokine receptor interaction** | | |  |  |  |
|  | M37435 | colony stimulating factor 1 (macrophage) | CSF1 | 3.6 | 0.00000 |
|  | BG170541 | met proto-oncogene (hepatocyte growth factor receptor) | MET | 4.2 | 0.00007 |
|  | U50748 | leptin receptor | LEPR | 6.1 | 0.00006 |
|  | NM_002193 | inhibin, beta B (activin AB beta polypeptide) | INHBB | 11.6 | 0.00001 |
|  | BE217880 | interleukin 7 receptor | IL7R | 13.8 | 0.00001 |
|  | BE568134 | tumor necrosis factor receptor superfamily, member 21 | TNFRSF21 | 27.6 | 0.00000 |
|  | NM_000641 | interleukin 11 | IL11 | 36.2 | 0.00001 |
| **Neuroactive ligand-receptor interaction** | | |  |  |  |
|  | NM_000824 | glycine receptor, beta | GLRB | 3.5 | 0.00072 |
|  | NM_001955 | endothelin 1 | EDN1 | 3.7 | 0.00020 |
|  | NM_004961 | gamma-aminobutyric acid (GABA) A receptor, epsilon | GABRE | 8.5 | 0.00016 |
|  | AI934556 | nuclear receptor subfamily 3, group C, member 1 | NR3C1 | 12.2 | 0.00010 |
|  | NM_006681 | neuromedin U | NMU | 15.9 | 0.00000 |
|  | AA897516 | prostaglandin E receptor 4 (subtype EP4) | PTGER4 | 23.1 | 0.00006 |
|  | BF055366 | endothelial differentiation, lysophosphatidic acid GPCR, 2 | EDG2 | 40.6 | 0.00029 |
|  | BE965369 | coagulation factor II (thrombin) receptor-like 1 | F2RL1 | 49.1 | 0.00000 |
