# Supplementary material for: BRCA1 Regulates Follistatin Function in Ovarian Cancer and Human Ovarian Surface Epithelial Cells
Source: PLoS One. 2012 Jun 1;7(6):e37697. doi: 10.1371/journal.pone.0037697 (PMC3365892; doi:10.1371/journal.pone.0037697)
Supplement: Table S3 — Pathway specific down-regulation of genes with the up-regulation of BRCA1 in SKOV3 cells. (DOC) [file pone.0037697.s005.doc]

| **Pathway**  **Table S3**: **Pathway specific down-regulation of genes with the up-regulation of BRCA1 in SKOV3 cells** | **Accession number** | **Gene name** | **Symbol** | **Folds Change** | **p-value** |
| --- | --- | --- | --- | --- | --- |
|  |  |  |  |  |  |
| **Metabolism** | | |  |  |  |
|  | N25727 | serine hydroxymethyltransferase 1 (soluble) | SHMT1 | -3.1 | 0.00001 |
|  | NM_000852 | glutathione S-transferase pi | GSTP1 | -3.2 | 0.00042 |
|  | AI656232 | OTU domain, ubiquitin aldehyde binding 2 | OTUB2 | -4.6 | 0.00072 |
|  | AL832250 | ubiquitin protein ligase E3A | UBE3A | -5 | 0.00007 |
|  | NM_002133 | heme oxygenase (decycling) 1 | HMOX1 | -5.5 | 0.00001 |
|  | AI635160 | trimethyllysine hydroxylase, epsilon | TMLHE | -6.7 | 0.00074 |
|  | NM_006169 | nicotinamide N-methyltransferase | NNMT | -6.8 | 0.00025 |
|  | AL556703 | ceruloplasmin (ferroxidase) | CP | -13.2 | 0.00001 |
|  | NM_014298 | quinolinate phosphoribosyltransferase | QPRT | -15.5 | 0.00023 |
|  | NM_001144 | autocrine motility factor receptor | AMFR | -24.6 | 0.00011 |
|  | BE613178 | cystathionine-beta-synthase | CBS | -46.3 | 0.00002 |
|  | BG120535 | vanin 1 | VNN1 | -68.1 | 0.00052 |
| **Basal transcription factors** | |  |  |  |  |
|  | AI377688 | general transcription factor IIH, polypeptide 1, 62kDa | GTF2H1 | -3.4 | 0.00005 |
|  | AF078847 | general transcription factor IIH, polypeptide 2, 44kDa | GTF2H2 | -3.9 | 0.00066 |
|  | AI366784 | TAF4b RNA polymerase II, TATA box binding protein (TBP)-associated factor, 105kDa | TAF4B | -4.2 | 0.00063 |
|  | D50544 | TAF12 RNA polymerase II, TATA box binding protein (TBP)-associated factor, 20kDa | TAF12 | -4.2 | 0.00063 |
| **MAPK signaling pathway** | |  |  |  |  |
|  | NM_004579 | mitogen-activated protein kinase kinase kinase kinase 2 | MAP4K2 | -3.4 | 0.00027 |
|  | NM_004313 | arrestin, beta 2 | ARRB2 | -4.2 | 0.00015 |
|  | D12625 | neurofibromin 1 | NF1 | -4.3 | 0.00066 |
|  | NM_002006 | fibroblast growth factor 2 (basic) | FGF2 | -6.8 | 0.00014 |
|  | NM_000594 | tumor necrosis factor (TNF superfamily, member 2) | TNF | -8.8 | 0.00018 |
|  | NM_005345 | heat shock 70kDa protein 1A | HSPA1A | -93.7 | 0.00025 |
| **Calcium signaling pathway** | | |  |  |  |
|  | AA401492 | GNAS complex locus | GNAS | -4.5 | 0.00058 |
|  | AI078167 | nuclear factor of kappa light polypeptide enhancer in B-cells inhibitor, alpha | NFKBIA | -4.7 | 0.00009 |
|  | AI625747 | adrenergic, beta-1-, receptor | ADRB1 | -5.4 | 0.00008 |
| **Jak-STAT signaling pathway** | | |  |  |  |
|  | AL039831 | Janus kinase 1 (a protein tyrosine kinase) | JAK1 | -3.1 | 0.00036 |
|  | NM_000640 | interleukin 13 receptor, alpha 2 | IL13RA2 | -6.1 | 0.00025 |
| **Wnt signaling pathway** | | |  |  |  |
|  | AI669212 | protein phosphatase 2, regulatory subunit B (PR 52), gamma isoform | PPP2R2C | -7.5 | 0.00002 |
|  | AB043703 | frizzled homolog 8 (Drosophila) | FZD8 | -8 | 0.00029 |
| **Notch signaling pathway** | | |  |  |  |
|  | AF029778 | jagged 2 | JAG2 | -3.4 | 0.00016 |
|  | NM_016941 | delta-like 3 (Drosophila) | DLL3 | -6.1 | 0.00014 |
| **Insulin signaling pathway** | | |  |  |  |
|  | NM_016203 | protein kinase, AMP-activated, gamma 2 non-catalytic subunit | PRKAG2 | -3.2 | 0.00017 |
| **Cytokine-cytokine receptor interaction** | | |  |  |  |
|  | NM_003811 | tumor necrosis factor (ligand) superfamily, member 9 | TNFSF9 | -3.5 | 0.0001 |
|  | L41944 | interferon (alpha, beta and omega) receptor 2 | IFNAR2 | -3.7 | 0.00003 |
|  | M60316 | bone morphogenetic protein 7 (osteogenic protein 1) | BMP7 | -6.1 | 0.00005 |
|  | NM_002993 | chemokine (C-X-C motif) ligand 6 (granulocyte chemotactic protein 2) | CXCL6 | -11.7 | 0.00029 |
|  | M57731 | chemokine (C-X-C motif) ligand 2 | CXCL2 | -15.3 | 0.00041 |
|  | NM_000584 | interleukin 8 | IL8 | -16.4 | 0.00006 |
| **Neuroactive ligand-receptor interaction** | | |  |  |  |
|  | NM_021077 | neuromedin B | NMB | -3.2 | 0.00001 |
|  | BC036030 | gamma-aminobutyric acid (GABA) A receptor, gamma 2 | GABRG2 | -5.7 | 0.00015 |
|  | AF056085 | G protein-coupled receptor 51 | GPR51 | -7.5 | 0.00066 |
|  | AL556409 | galanin | GAL | -12.8 | 0.00001 |
| **Cell cycle** |  |  |  |  |  |
|  | AI950069 | transcription factor Dp-1 | TFDP1 | -3.9 | 0.00001 |
|  | NM_000077 | cyclin-dependent kinase inhibitor 2A (melanoma, p16, inhibits CDK4) | CDKN2A | -21.4 | 0.00006 |
|  | NM_001759 | cyclin D2 | CCND2 | -122.5 | 0.00031 |
| **Cell adhesion molecules (CAMs)** | | |  |  |  |
|  | AI762627 | protein tyrosine phosphatase, receptor type, F | PTPRF | -3.6 | 0.00024 |
|  | AA149644 | junctional adhesion molecule 3 | JAM3 | -7.9 | 0.00003 |
|  | NM_001078 | vascular cell adhesion molecule 1 | VCAM1 | -10.9 | 0.00003 |
|  | BF218922 | chondroitin sulfate proteoglycan 2 (versican) | CSPG2 | -12.4 | 0.00014 |
|  | NM_006580 | claudin 16 | CLDN16 | -13.2 | 0.00002 |
|  | AI608725 | intercellular adhesion molecule 1 (CD54), human rhinovirus receptor | ICAM1 | -17.7 | 0.00013 |
|  | AW264204 | claudin 11 (oligodendrocyte transmembrane protein) | CLDN11 | -18.6 | 0.0002 |
|  | AF101051 | claudin 1 | CLDN1 | -22.5 | 0.00013 |
|  | NM_000201 | intercellular adhesion molecule 1 (CD54), human rhinovirus receptor | ICAM1 | -22.8 | 0.00019 |
| **Focal adhesion** | | |  |  |  |
|  | AA564926 | parvin, gamma | PARVG | -5.1 | 0.00009 |
|  | AA187563 | parvin, beta | PARVB | -13.3 | 0.00003 |
|  | NM_000393 | collagen, type V, alpha 2 | COL5A2 | -79 | 0.00015 |
| **Regulation of actin cytoskeleton** | | |  |  |  |
|  | NM_006633 | IQ motif containing GTPase activating protein 2 | IQGAP2 | -3.3 | 0.00044 |
|  | AL161999 | cytoplasmic FMR1 interacting protein 2 | CYFIP2 | -3.9 | 0.00015 |
| **Apoptosis** |  |  |  |  |  |
|  | AI246590 | interleukin-1 receptor-associated kinase 2 | IRAK2 | -5.1 | 0.00001 |
|  | U37546 | baculoviral IAP repeat-containing 3 | BIRC3 | -57.4 | 0.00012 |
